# Development of novel salicylic acid–donepezil–rivastigmine hybrids as multifunctional agents for the treatment of Alzheimer’s disease

**DOI:** 10.1080/14756366.2023.2231661

**Published:** 2023-07-06

**Authors:** Yi Zhou, Ying He, Xue Teng, Jing Mi, Jing Yang, Rongrui Wei, Wenmin Liu, Qinge Ma, Zhenghuai Tan, Zhipei Sang

**Affiliations:** a College of Chemistry and Pharmaceutical Engineering, Nanyang Normal University, Nanyang, China; b Key Laboratory of Tropical Biological Resources of Ministry of Education and One Health Institute, School of Pharmaceutical Sciences, Hainan University, Haikou, China; c Key Laboratory of Modern Preparation of Traditional Chinese Medicine of Ministry of Education, Research Center of Natural Resources of Chinese Medicinal Materials and Ethnic Medicine, Jiangxi University of Traditional Chinese Medicine, Nanchang, China; d Institute of Traditional Chinese Medicine Pharmacology and Toxicology, Sichuan Academy of Chinese Medicine Sciences, Chengdu, China

**Keywords:** Alzheimer’s disease, salicylic acid–donepezil–rivastigmine hybrids, multi-functional agent, drug-likeness, scopolamine-induced AD model

## Abstract

Alzheimer’s disease (AD) is a chronic, progressive brain degenerative disease that is common in the elderly. So far, there is no effective treatment. The multi-target-directed ligands (MTDLs) strategy has been recognised as the most promising approach due to the complexity of the pathogenesis of AD. Herein, novel salicylic acid–donepezil–rivastigmine hybrids were designed and synthesised. The bioactivity results exhibited that **5a** was a reversible and selective *eq*BChE inhibitor (IC_50_ = 0.53 μM), and the docking provided the possible mechanism. Compound **5a** also displayed potential anti-inflammatory effects and significant neuroprotective effect. Moreover, **5a** exhibited favourable stabilities in artificial gastrointestinal solution and plasma. Finally, **5a** demonstrated potential cognitive improvement in scopolamine-induced cognitive dysfunction. Hence, **5a** was a potential multifunctional lead compound against AD.

## Graphical Abstract

**Figure:**
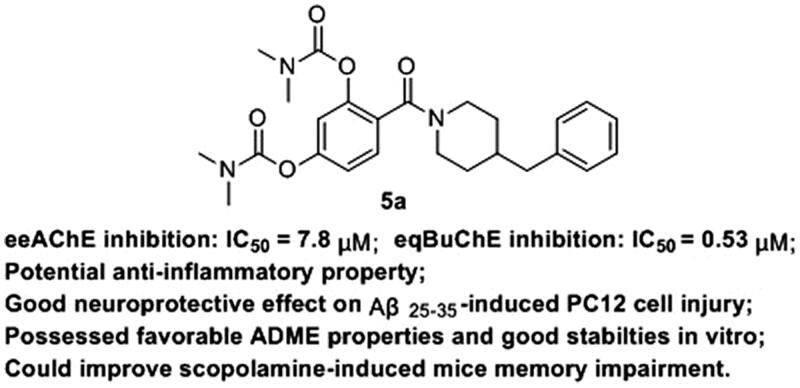

## Introduction

Alzheimer’s disease (AD) is a chronic and progressive neurodegenerative disease of the brain that commonly occurs in the elderly^1^. So far, more than 55 million people worldwide are estimated to have dementia, and by 2050, the figure will rise to 139 million^2^. Currently, there is no known cure for AD or alters the disease process in the brain^3^.

Although many efforts have been made to study AD, the exact pathogenesis is still not clear. Several abnormal brain changes (including amyloid-*β* (A*β*) aggregations, levels of acetylcholine (ACh) decreased and neuroinflammation) have been suggested to serve as important roles in the pathogenesis of AD. Thus, the multi-target-directed ligands (MTDLs) approach has been recognised and developed as the most promising approach due to the complex pathogenesis^4–6^.

Based on the cholinergic hypothesis, three AChE inhibitors (donepezil, galanthamine, and rivastigmine) have been marketed to treat AD by recovering ACh levels. While, long-term clinical uses indicate that the above drugs can only locally relieve the symptoms of AD, and the inhibition of peripheral AChE leads to nausea, dizziness, and vomiting^7^. Furthermore, as AD progressed, AChE levels in the AD brains sharply decreased by 90%, while the levels of BuChE increased to 165% compared with normal levels, indicating that BuChE takes over AChE to hydrolyse ACh in progressive AD^8^. Therefore, inhibition of BuChE could be a potential anti-AD approach.

The amyloid hypothesis states that the production and accumulation of A*β* is the key pathogenesis leading to AD^9^. A*β* oligomeric aggregates show remarkable neurotoxicity. Moreover, A*β* is able to enter mitochondria where it increases ROS production and induces oxidative stress, and further damages biological macromolecules such as lipids, DNA, and proteins. Furthermore, neuroinflammation is also a major pathogenesis in AD patients and is associated with astrocytes, microglia, and inflammatory factors^10^. Investigations have shown that ADf patients have significantly increased levels of proinflammatory cytokines (such as IL-6, TNF-α, and IL-1*β*) in serum and brain, which further increase A*β* deposition and lead to cognitive dysfunction and neuron loss^11^^,^^12^. Hence, anti-inflammation is an effective treatment for AD.

**Figure 1.**
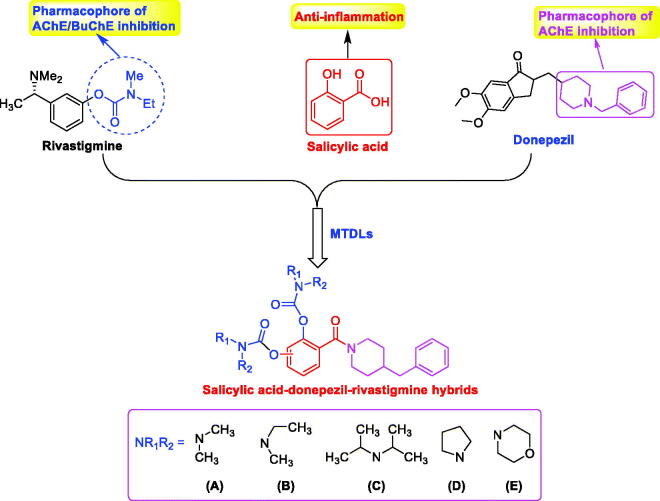
Design of salicylic acid–donepezil–rivastigmine hybrids.

Salicylic acid (SA) is a non-steroidal anti-inflammatory drug found mainly in vegetables and fruits. The study shows that SA exhibits weak anticholinesterase activity (IC_50_ = 346.0 μM)^13^. As we all known, 1-benzylpiperidine of donepezil is the key pharmacophore, which has been applied for the development of novel ChEs inhibitors^4^^,^^14^. In our previous work, 1-benzylpiperidine has been introduced into SA to develop as BuChE inhibitor^15^. In addition, rivastigmine is an FDA-approved dual AChE/BuChE inhibitor and the carbamate fragment is the pharmacophore, and many selective BuChE inhibitors have been developed by introducing carbamate fragment^16–19^. Therefore, based on the SA skeleton, novel SA–donepezil–rivastigmine hybrids were designed and synthesised by the simultaneous introduction of benzylpiperidine and carbamate fragments (Figure 1), hoping the optimised compounds possess multiple properties including BuChE inhibition, anti-inflammation, and neuroprotective effects.

## Results and discussion

### Chemistry

**Scheme 1.**
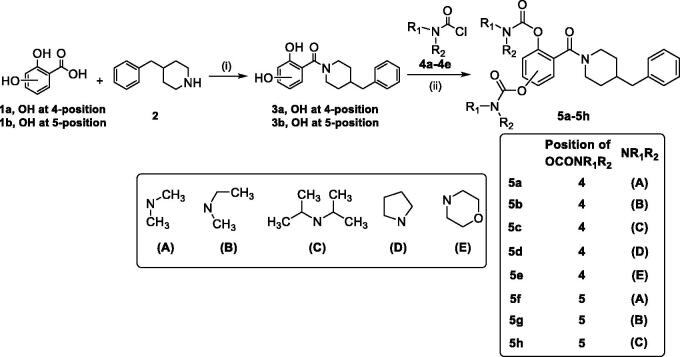
Synthesis of **5a**–**5h**. Conditions: (i) EDCI, HOBT, THF, and room temperature. (ii) *N*,*N*-disubstituted carbamoyl chlorides (**4a**–**4e**), CH_3_CN, 65 °C.

The synthetic route of target compounds **5a**–**5h** is described in Scheme 1. Briefly, the starting materials **1a**–**1b** (2,4-dihydroxybenzoic acid **1a**; 2,5-dihydroxybenzoic acid **1b**) were reacted with 4-benzylpiperidine (**2**) in the presence of EDCI and HOBT in THF to gain compounds **3a** and **3b**. Finally, compound **3a** or **3b** was reacted with excessive amounts of *N*,*N*-disubstituted carbamoyl chlorides (**4a**–**4e**), respectively, to afford target compounds **5a**–**5h**.

### Biological activity

#### Inhibition effects of target compounds on AChE and BuChE

The *ee*AChE (from *electric eel*)/*eq*BuChE (from *equine serum*) inhibitory activity of compounds **5a**–**5h** were evaluated through Ellman’s method^20^^,^^21^. Donepezil and rivastigmine were also evaluated as control compounds. The results are displayed in Table 1, overall speaking, **5a**–**5h** demonstrated significant AChE/BuChE inhibitory potency compared with the starting material **1a** and **1b**, suggesting that the introduction of carbamate fragments into SA skeleton could improve AChE/BuChE inhibitory effects. **5a**–**5f** exhibited significant *eq*BuChE inhibitory activity (IC_50_ values ranging from 0.53 μM to 13.1 μM) and potent *ee*AChE inhibitory activity (IC_50_ was ranging from 6.3 μM to 22.3 μM). The introduction of carbamate fragment increased the AChE/BuChE inhibition. **5a** with dimethylcarbamoyl fragment indicated good *ee*AChE inhibitory potency (IC_50_ = 7.8 μM). When the dimethylcarbamate fragment of **5a** was replaced with the *N*-ethyl-*N*-methylcarbamate fragment to obtain **5b**, the *ee*AChE inhibitory capacity was 6.3 μM, when changing dimethylcarbamoyl fragment of **5a** with diisopropylcarbamoyl fragment to get **5c** (IC_50_ = 7.1 μM), no significant change in *ee*AChE inhibitory potency was produced. In contrast, when the dimethylcarbamoyl fragment of **5a** was displaced with 1-pyrrolidinecarbonyl and 4-morpholinecarbonyl fragment to obtain **5d** and **5e**, respectively, the inhibitory capacity of *ee*AChE sharply reduced to 22.3 μM and 18.3 μM, respectively, suggesting that 1-pyrrolidinecarbonyl and 4-morpholinecarbonyl fragment negatively affected the *ee*AChE inhibitory potency. Furthermore, compound **5a** showed excellent *eq*BuChE inhibitory potency with IC_50_ value of 0.53 μM, when the dimethylcarbamoyl fragment of **5a** was replaced with *N*-ethyl-*N*-methylcarbamoyl fragment to get **5b**, the *eq*BuChE inhibitory potency significantly decreased to 2.6 μM, when the dimethylcarbamoyl fragment of **5a** was replaced with diisopropylcarbamoyl, 1-pyrrolidinecarbonyl, and 4-morpholinecarbonyl fragment to afford compounds **5c**–**5e**, the *eq*BuChE sharply reduced to 10.8 μM, 8.7 μM, and 13.1 μM, respectively. Further, the position of carbamate fragment also influenced the inhibitory potency of *ee*AChE and *eq*BuChE. When the carbamate fragment at 4-position was displaced with carbamate fragment at 5-position to afford compounds **5f**–**5h**, it showed significant *ee*AChE inhibitory capacity (IC_50_ values ranging from 4.9 μM to 8.2 μM) and weak *eq*BuChE inhibitory activity (IC_50_ was ranging from 13.5 μM to 16.7 μM). Compared with compound **5a**, compound **5f** showed significant *ee*AChE inhibitory capacity (IC_50_ = 5.2 μM) and weak *eq*BuChE inhibitory capacity (IC_50_ = 16.3 μM). **5g** demonstrated a potential *ee*AChE inhibition capacity (IC_50_ = 4.9 μM) and a sharp decrease in *eq*BuChE to 13.5 μM compared to **5b**. Compound **5h** showed similar *ee*AChE inhibitory potency (IC_50_ = 8.2 μM) and *eq*BuChE inhibitory capacity (IC_50_ = 16.7 μM) compared with compound **5c**, respectively. Totally speaking, **5a** was a promising *eq*BuChE inhibitor, deserving further investigation.

**Table 1.** The inhibitory capacity of AChE/BuChE by **5a**–**5h**, rivastigmine and donepezil.

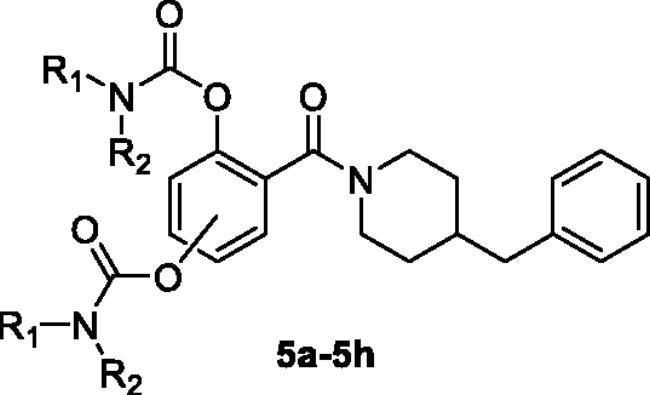					
Comp.	Position of OCONR_1_R_2_	NR_1_R_2_	IC_50_ ± SD^a^ (μM)		SI^d^
			*ee*AChE^b^	*eq*BuChE^c^	
**5a**	4	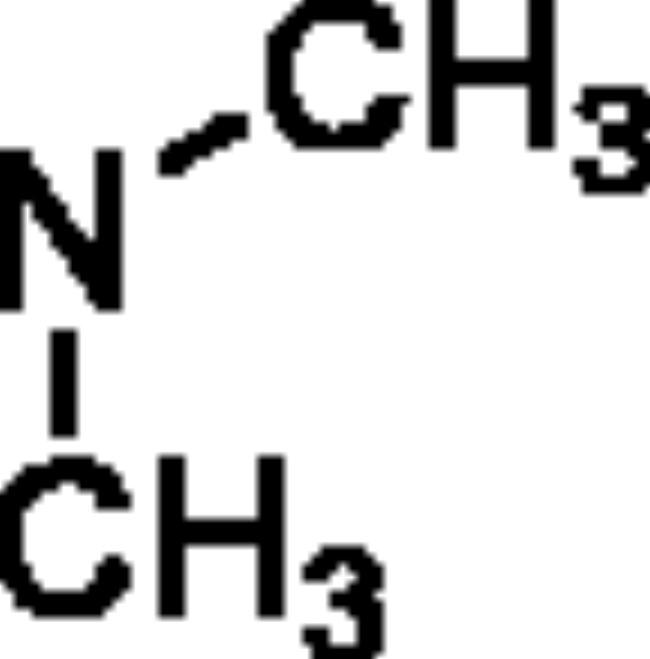	7.8 ± 0.36	0.53 ± 0.01	14.7
**5b**	4	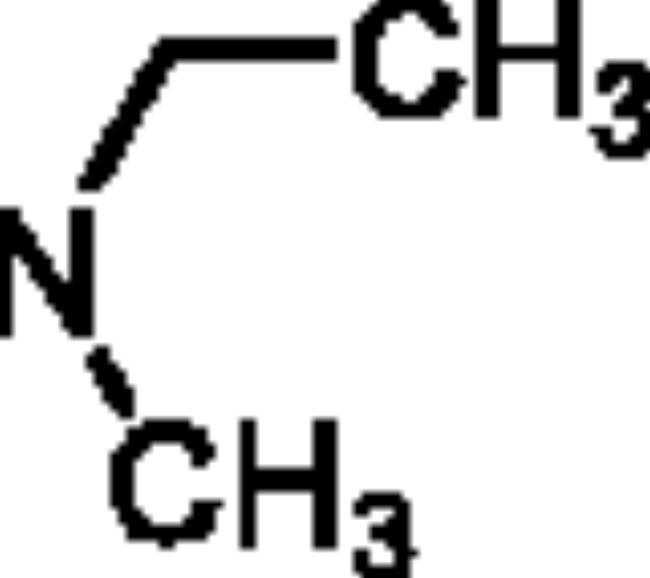	6.3 ± 0.41	2.6 ± 1.8	2.4
**5c**	4	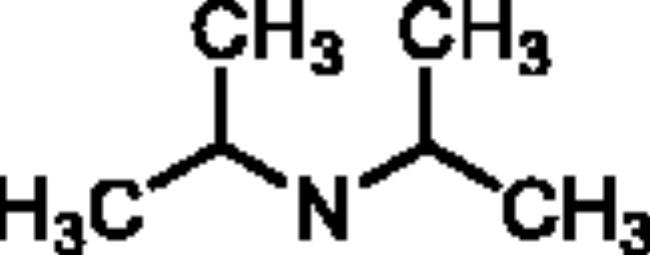	7.1 ± 0.23	10.8 ± 0.56	0.66
**5d**	4	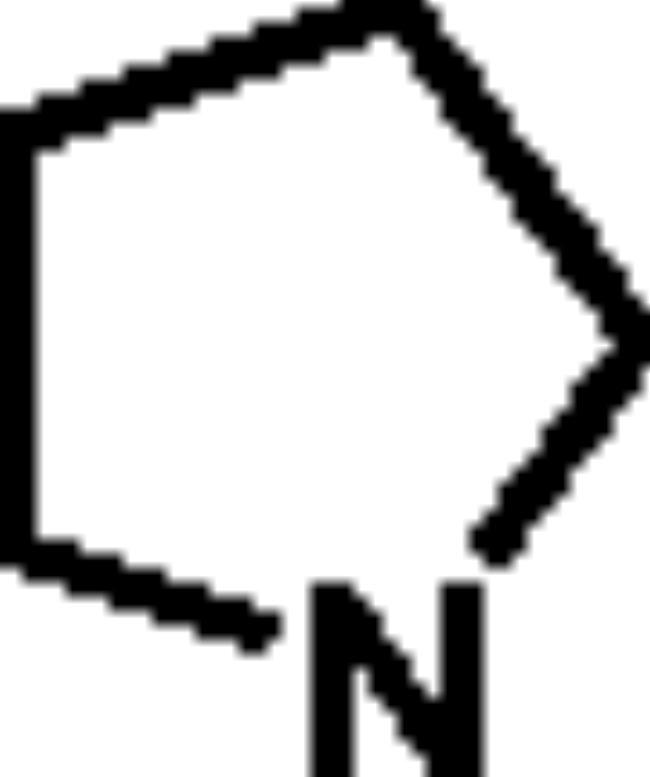	22.3 ± 1.8	8.7 ± 0.93	2.6
**5e**	4	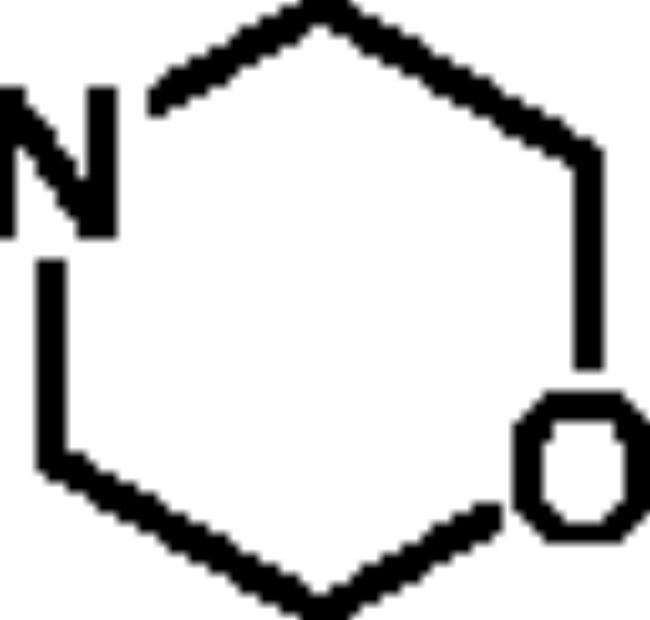	18.3 ± 0.87	13.1 ± 0.72	1.4
**5f**	5	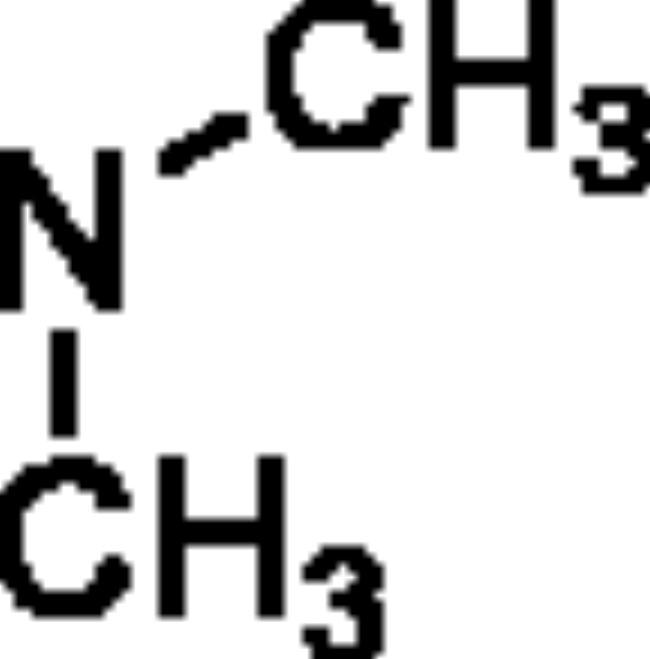	5.2 ± 0.35	16.3 ± 0.86	0.32
**5g**	5	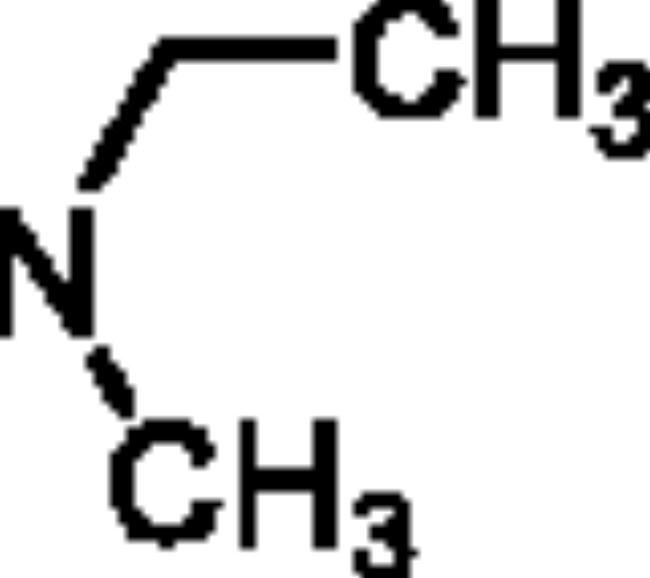	4.9 ± 0.68	13.5 ± 0.52	0.36
**5h**	5	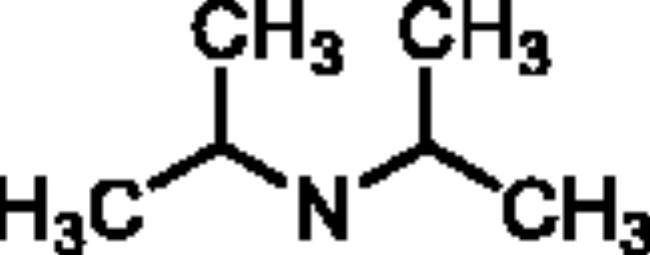	8.2 ± 0.67	16.7 ± 1.2	0.49
**1a**			16.3 ± 0.17%^e^	25.8 ± 0.93%^e^	–
**1b**			20.6 ± 1.2%^e^	18.2 ± 0.67%^e^	–
**Donepezil**			0.018 ± 0.002	7.6 ± 0.68	0.003
**Rivastigmine**			11.3 ± 0.08	4.9 ± 0.02	2.3

^a^ IC_50_ was indicated as the mean ± SD.

^b^ *ee*AChE = *electric eel* AChE.

^c^ *eq*BChE = *equine serum* BuChE.

^d^ SI = IC_50_ (AChE)/IC_50_ (BuChE).

^e^ Inhibition percent rate at 25 μM.

#### The reversibility of BuChE inhibition by 5a

**Figure 2.**
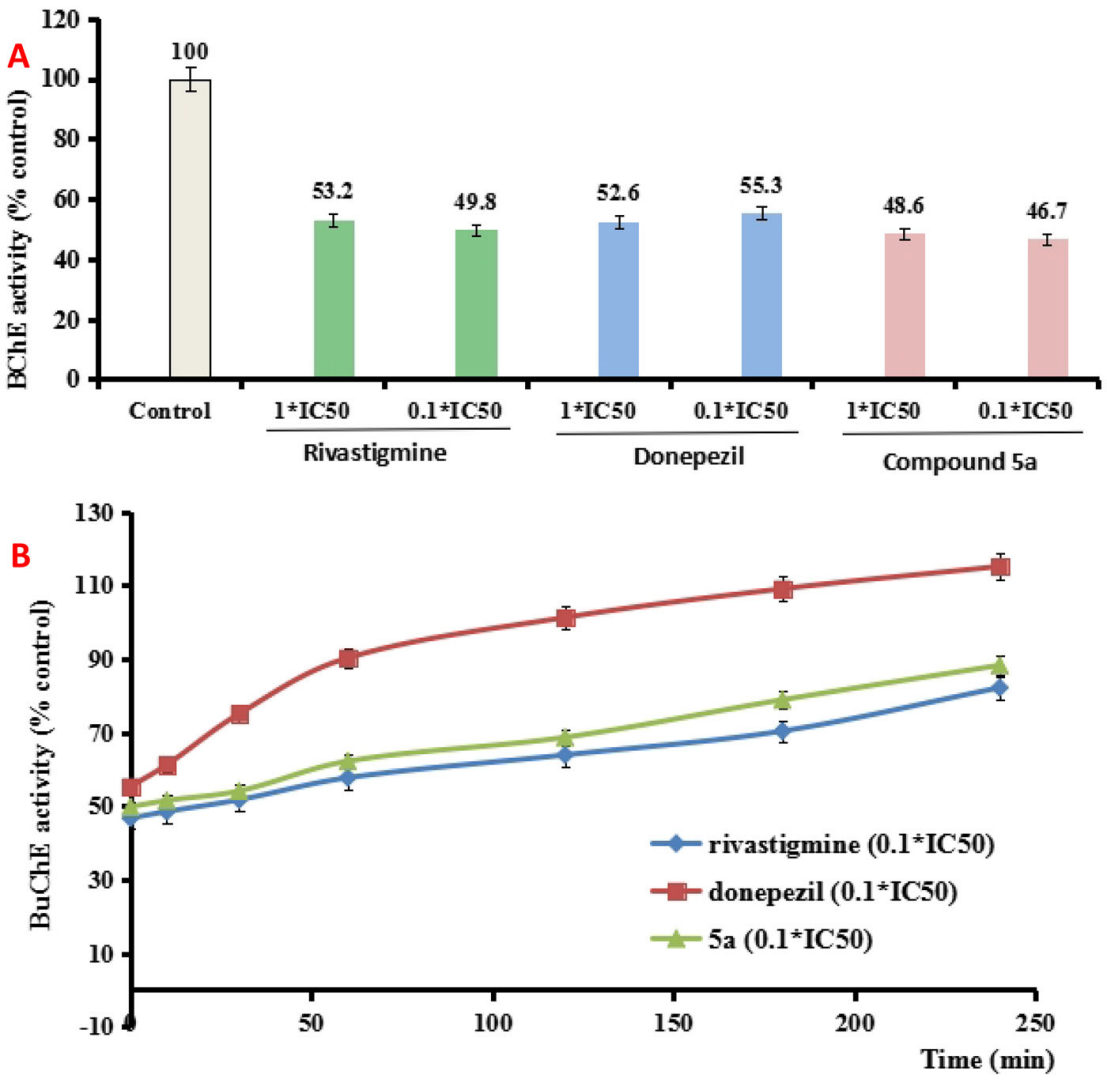
(A) BuChE recovery capability after preincubation of compound **5a** was diluted to 0.1 × IC_50_. (B) The recovery capability of BuChE inhibition by rivastigmine, donepezil, and **5a** was diluted to 0.1 × IC_50_ by monitoring with time for 240 min. The results were indicated as the mean ± SEM.

To determine whether **5a** was a reversible BuChE inhibitor, we evaluated the recovery capability of *eq*BuChE inhibitors inhibition after dilution to 0.1 × IC_50_, rivastigmine and donepezil were also evaluated as control compounds^22^. As exhibited in Figure 2(A), the *eq*BuChE activity restored to 2.7% when donepezil was diluted to 0.1 × IC_50_, while no significant recovery of *eq*BuChE activity occurred when rivastigmine and **5a** was diluted to 0.1 × IC_50_, respectively. Further, the recovery capability of BuChE inhibitory effect after dilution was monitored by time. As indicated in Figure 2(B), donepezil at 0.1 × IC_50_ recovered BuChE activity to 101.3% at 120 min, and rivastigmine at 0.1 × IC_50_ gradually restored BuChE activity to 68.7% at 120 min, suggesting that donepezil was a reversible *hu*AChE inhibitor and rivastigmine was a pseudo-irreversible *hu*AChE inhibitor. Under the same conditions, the 0.1 × IC_50_ **5a** gradually restored BuChE activity to 63.9% at 120 min, which was similar with rivastigmine. Therefore, the data showed that **5a** was a pseudo-irreversible BuChE inhibitor.

#### Docking study

**Figure 3.**
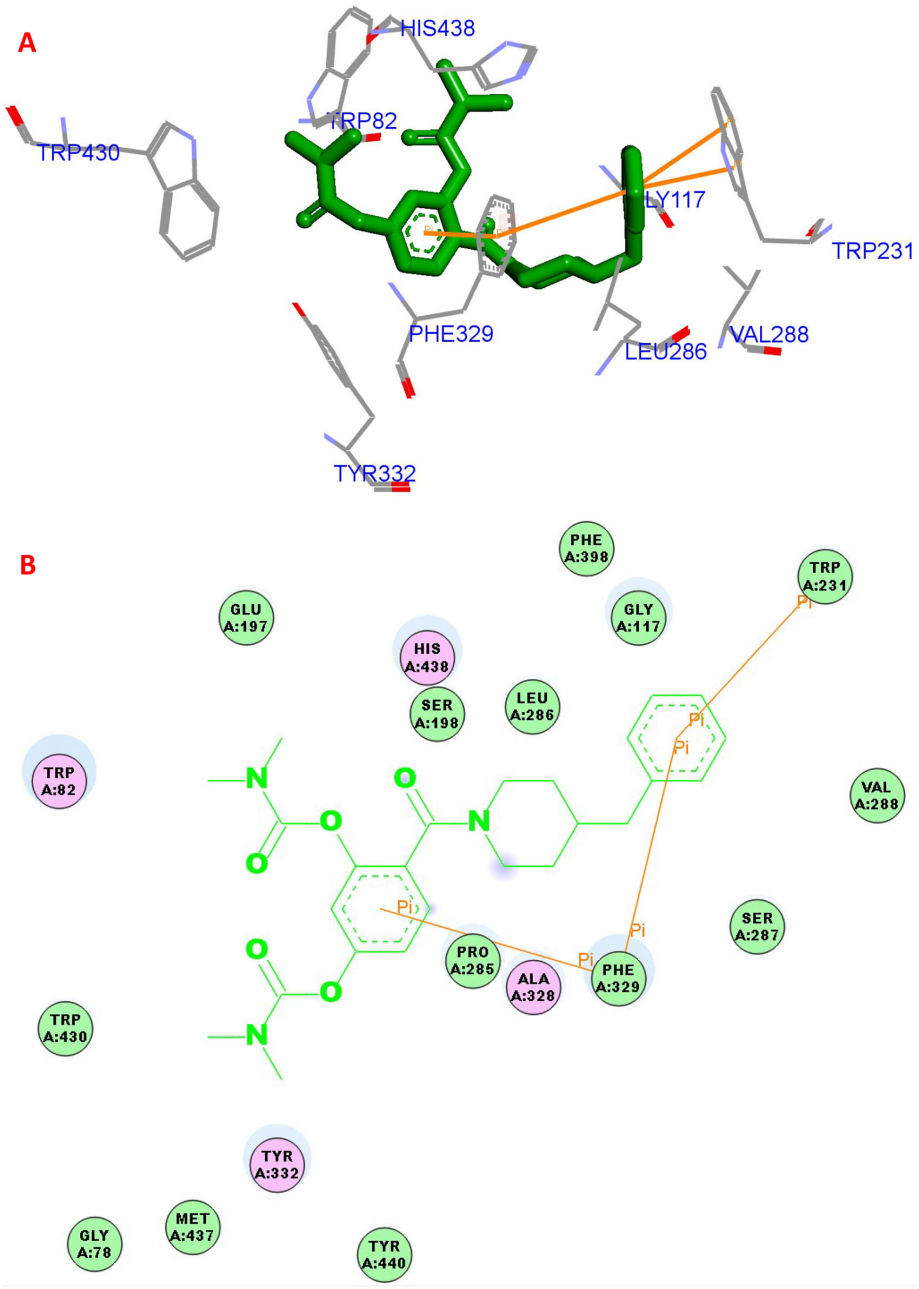
(A) **5a** (green stick) interacted with the binding residues of *hu*BuChE (PDB code: 4tpk). (B) 2D interaction of **5a** (green stick) with *hu*BuChE.

The possible binding action of **5a** with *hu*BChE (PDB code: 4tpk) was carried out by molecular docking^20^^,^^23^^,^^24^. In the **5a**−*hu*BuChE complex (Figure 3(A,B)), the benzene ring of SA formed one π–π interaction (5.0 Å) with key residue Phe329, the benzene ring of 4-benzylpiperidine formed one π–π interaction (5.8 Å) with key amino acid residue Phe329. The benzene ring of 4-benzylpiperidine formed two π–π interactions (3.8 Å and 4.8 Å, respectively) with key amino acid residue Trp231. Furthermore, hydrophobic interactions were also found between **5a** and residues Trp82, Phe329, His438, Trp231, Leu286, Gly117, Val288, and Trp430. Thus, the multi-interactions of *hu*BuChE−**5a** complex might provide possible mechanism for the high BuChE inhibitory capability.

#### Anti-inflammation of 5a

**Figure 4.**
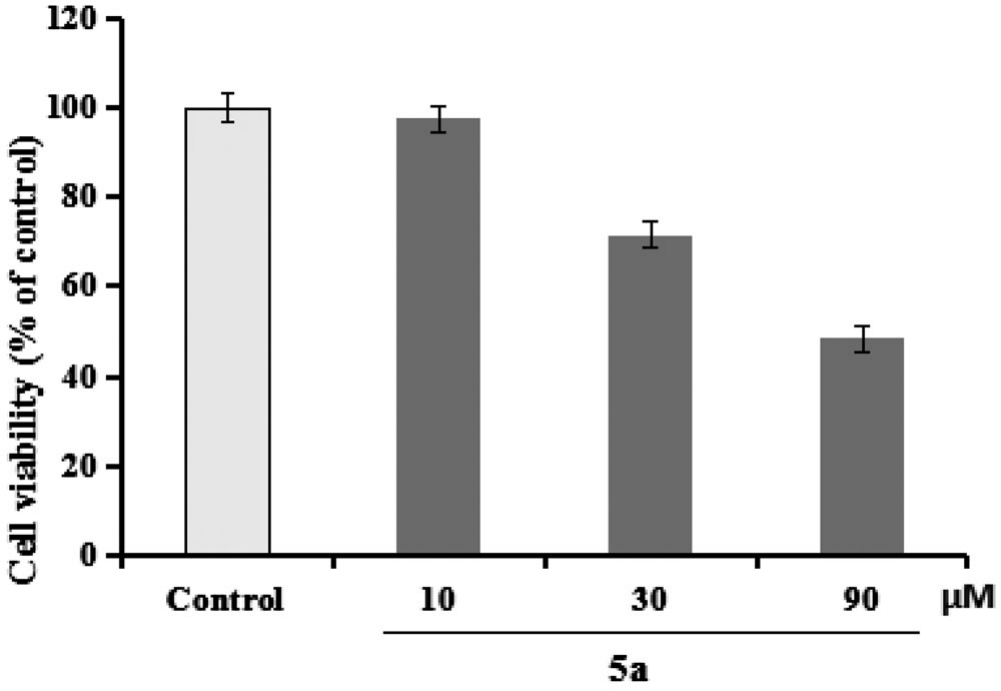
The cell viability was determined using CCK-8 assay. The results were indicated as the mean ± SD.

**5a** was chosen to evaluate the anti-inflammation property through testing the production of IL-1β, IL-6, and NO in LPS-induced BV-2 cells^25^^,^^26^. First of all, the cytotoxicity of **5a** was determined through the CCK-8 assay. As demonstrated in Figure 4, compound **5a** did not produce significant cytotoxicity on BV-2 cells at 10 μM.

##### Evaluation of the production of IL-6, IL-1β, and NO in LPS-stimulated BV-2 cells

**Figure 5.**
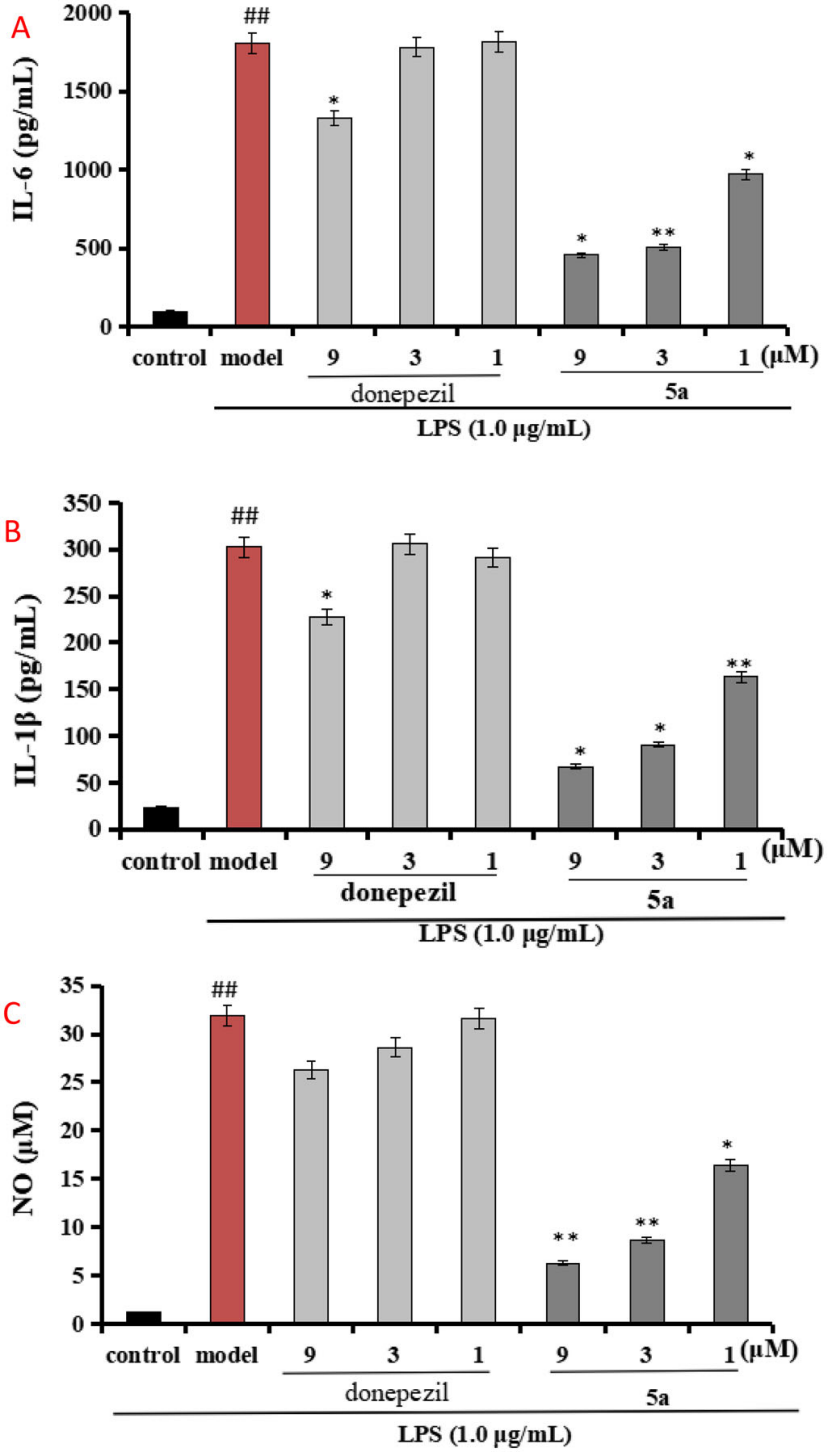
(A) Effects of compounds **5a** and donepezil on the production of IL-6; (B) effects of compound **5a** and donepezil on the production of IL-1β; (C) effects of compound **5a** and donepezil on NO release. The results were expressed as the mean ± SD. *p < 0.05, **p < 0.01 vs. model group; ^##^*p* < 0.01 vs. control.

Compound **5a** was selected to evaluate the anti-inflammation property, and the positive drug donepezil was also tested as comparison purpose. As displayed in Figure 5(A), when BV-2 cells were treated with LPS (1 μg/mL), the level of IL-6 significantly elevated to 1805.2 pg/mL (*p* < 0.01) compared with normal group (100.5 pg/mL), when treating with **5a** (1, 3, and 9 μM), the levels of IL-6 remarkably declined to 456.2 pg/mL, 505.3 pg/mL, and 971.1 pg/mL, respectively. Furthermore, Figure 5(B) shows that the levels of IL-1β significantly elevated to 302.7 pg/mL (*p* < 0.01) after the addition of 1 μg/mL LPS, when treating with **5a** (1, 3, and 9 μM), the levels of IL-1β remarkably declined to 67.2 pg/mL, 90.9 pg/mL, and 163.4 pg/mL, respectively. Moreover, as demonstrated in Figure 5(C), when BV-2 cells were treated with LPS (1 μg/mL), the release volume of NO remarkably elevated to 31.9 μM (*p* < 0.01), when treating with **5a** (1, 3, and 9 μM), the release volume of NO remarkably declined. The data showed that **5a** exhibited good anti-inflammatory activity compared with the positive drug donepezil.

#### Neuroprotective effects of 5a

**Figure 6.**
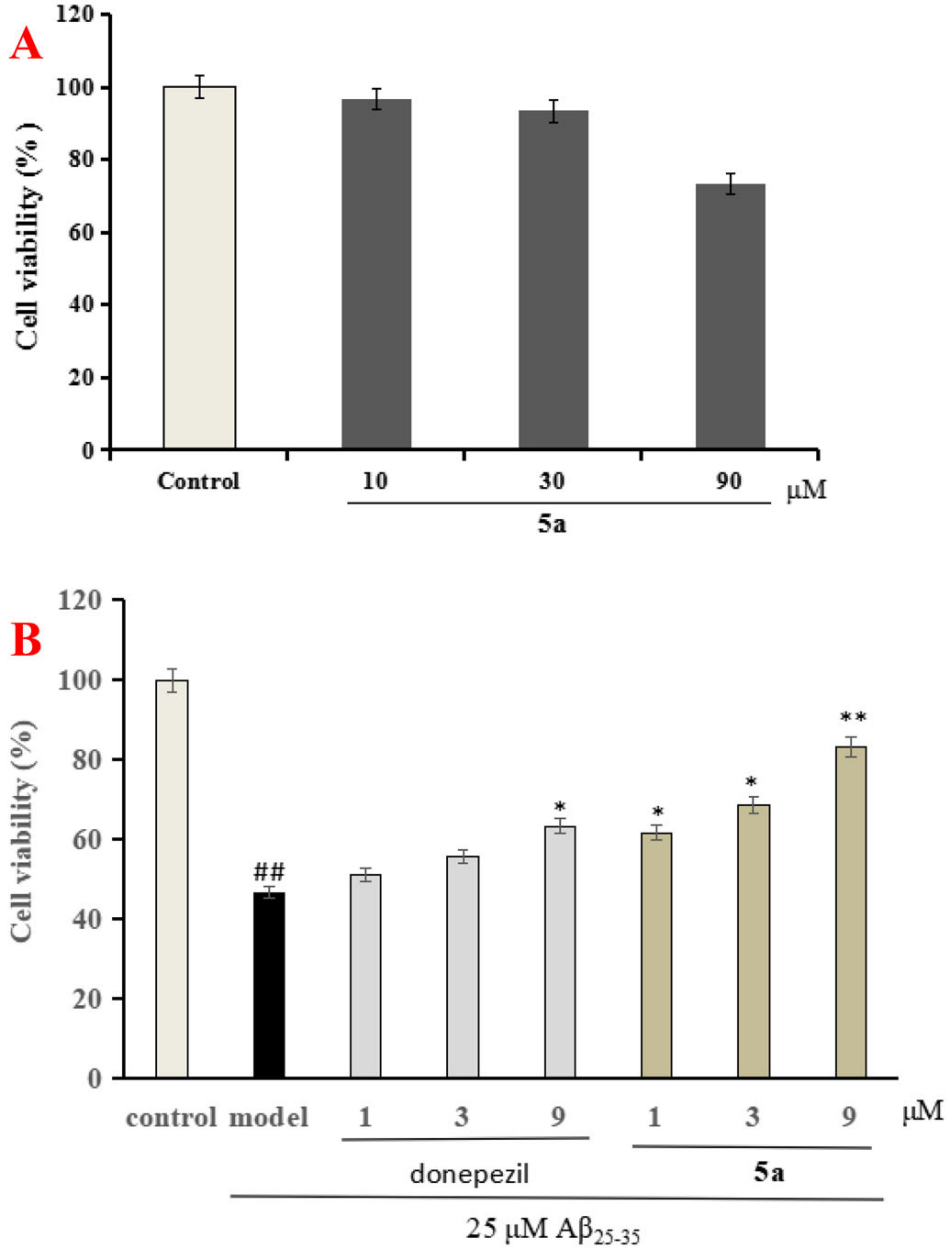
(A) The cytotoxicity of **5a**. (B) Neuroprotective effects of compounds **5a** and donepezil on A*β*_25-35_-induced PC12 cell injury by CCK-8 assay. Data were listed as the mean ± SD. *p < 0.05, **p < 0.01 vs. A*β*_25-35_-induced group; ^##^*p* < 0.01 vs. control group.

The CCK-8 assay was applied to assess the neuroprotective effect of **5a** on A*β*_25-35_-induced injury in PC12 cell, and donepezil was also tested as comparison purpose^27^^,^^28^. Figure 6(A) shows that compound **5a** shows no obvious cytotoxicity on PC12 cells at 30 μM. In addition, as demonstrated in Figure 6(B), donepezil showed potent neuroprotective effect at 9 μM, and when PC12 cells were exposed to 25 μM A*β*_25-35_ aggregates, the cell viability was reduced to 46.7% (*p* < 0.01). When treatment with **5a** (1, 3, and 9 μM), the PC12 cells viability improved to 61.7% (*p* < 0.05), 68.6% (*p* < 0.01), and 83.3% (*p* < 0.01), respectively. The data demonstrated that compound **5a** presented remarkable neuroprotective effects on A*β*_25-35_-induced PC12 cell injury compared with donepezil.

#### Prediction of drug-likeness

The QikProp module from Schröginger molecular modelling software was chosen to calculate the ADME property of **5a**^4^. The predictive indicators include molecular weight, hydrogen bond donors, hydrogen bond acceptors, polar surface area, hydrophilicity, permeability, and human oral absorption parameters. As demonstrated in Table S1, compound **5a** complied with both Lipinski’s rule of five. To sum up, compound **5a** demonstrated good drug-like properties.

#### Stability studies of compound 5a

**Figure 7.**
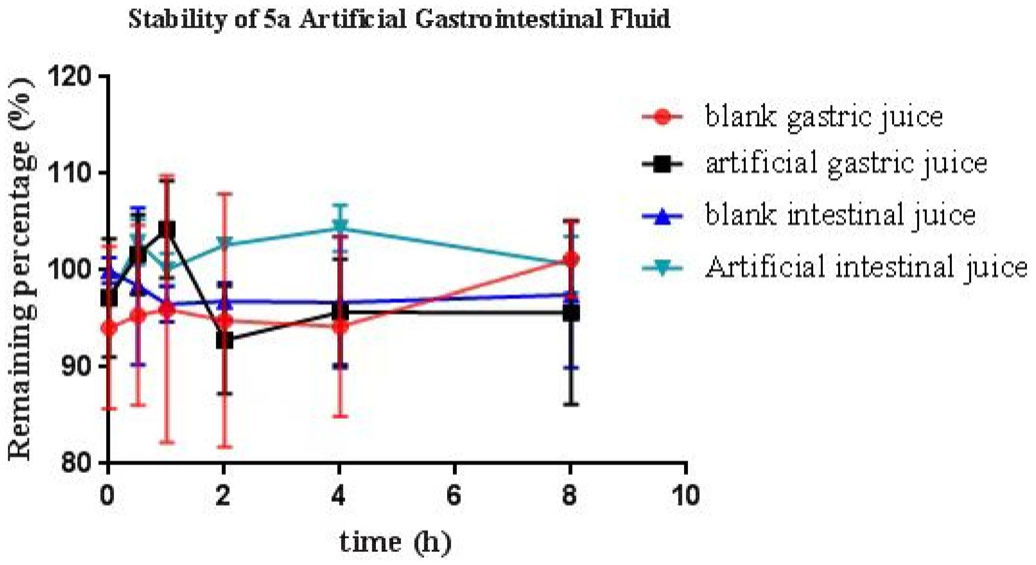
Stabilities of **5a** in artificial gastrointestinal fluids (*n* = 3).

Based on the above data, **5a** was chosen to evaluate the stabilities by artificial gastrointestinal solution, blood plasma, and rat liver microsomes^29^. As demonstrated in Figure 7, the residual amount of **5a** was 99.5%, 95.6%, 97.5%, and 110.6% after 8 h incubation in blank gastric juice, artificial gastric juice, blank intestinal juice, and artificial intestinal juice, respectively. The data suggested that **5a** was stable in artificial intestinal and gastric fluids.

**Figure 8.**
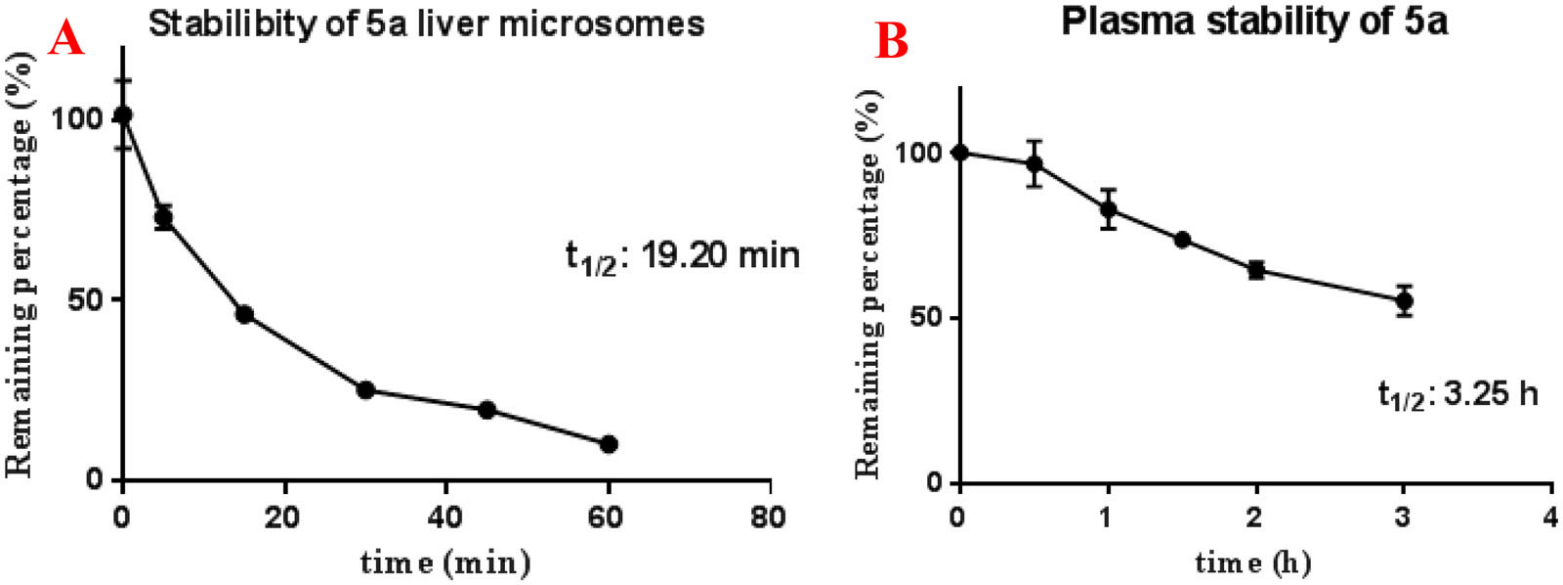
(A) Rat microsomes stability of **5a** (*n* = 3); (B) plasma stability of **5a** (*n* = 3).

Moreover, Figure 8 shows that compound **5a** displayed moderate stability in rat liver microsomes (*t*_1/2_ = 19.20 min) and favourable stability in blood plasma (*t*_1/2_ = 3.25 h).

#### Effects of compound 5a on scopolamine-induced cognitive dysfunction in mice

The effects of **5a** on scopolamine-induced cognitive dysfunction in mice were accessed by the step-down passive avoidance task^21^^,^^22^. First, the safety profile of **5a** was determined by Kunming mice (18–22 g, eligibility certification no. SCXK-Xiang2019-0004) at doses of 500 and 250 mg/kg through intragastric administration. The results showed that the mice did not present abnormal behaviour at the dose of 500 mg/kg.

**Figure 9.**
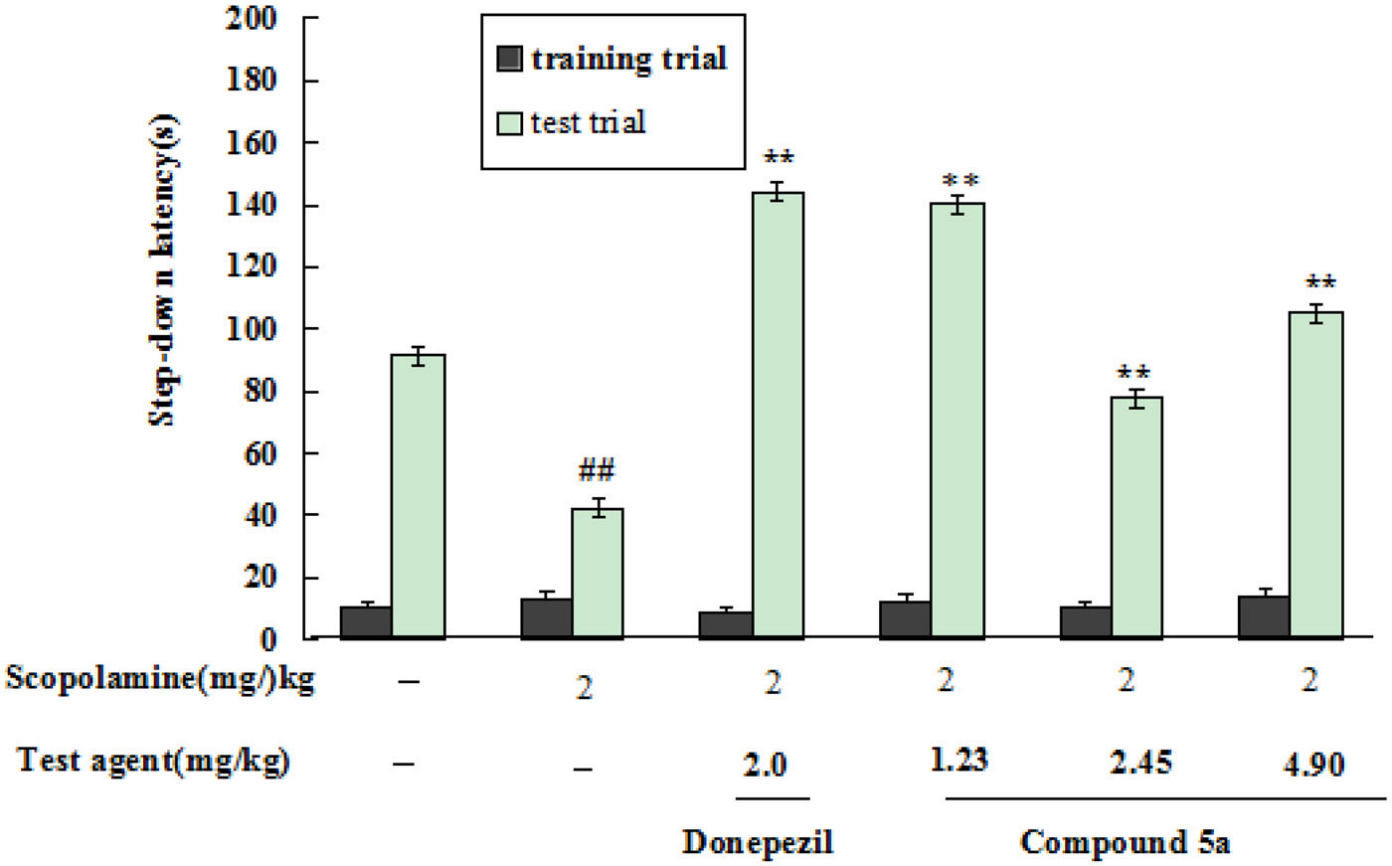
The *in vivo* effects of compound **5a** and donepezil on scopolamine-induced cognitive dysfunction in mice. The results were indicated as the mean ± SD. **p < 0.01 vs. scopolamine-induced group; ^##^*p* < 0.01 vs. normal group.

The *in vivo* effects of **5a** were determined through the step-down passive avoidance task. As demonstrated in Figure 9, when mice was given scopolamine at a dose of 2.0 mg/kg, the step-down latency remarkably reduced to 42.1 s (*p* < 0.01). When treatment with donepezil at a dose of 2.0 mg/kg, the step-down latency sharply increased to 144.3 s (*p* < 0.01). When treating with **5a** (1.23, 2.45, and 4.9 mg/kg), the step-down latency elevated to 140.1 s (*p* < 0.01), 77.8 s (*p* < 0.01), and 105.2 s (*p* < 0.01), respectively, suggesting that **5a** produced a precognitive effect on scopolamine-induced cognitive dysfunction in mice at a dose of 1.23 mg/kg.

## Conclusions

To date, there are no effective drugs capable of halting or reversing the progression of AD. As the complex pathogenesis of AD, MTDLs approach is regarded as the potential treatment. Thus, novel SA–donepezil–rivastigmine hybrids were designed and synthesised by the simultaneous introduction of benzylpiperidine and carbamate fragments. The results demonstrated that **5a** was a reversible and selective *eq*BChE inhibitor (IC_50_ = 0.53 μM). **5a** demonstrated potential anti-inflammation property by inhibiting the level of IL-6, IL-1β, and NO production, and significant neuroprotective effect on A*β*_25-35_-induced PC12 cell injury. Moreover, **5a** exhibited favourable stabilities in artificial gastrointestinal fluids, liver microsomes, and plasma. Finally, **5a** produced a precognitive effect on scopolamine-induced cognitive dysfunction in mice. All in all, **5a** was a potential multi-targeted anti-AD compound.

## Experimental

### Chemistry

All the chemicals and solvents were from commercial companies. The reactions were monitored by TLC, and crude products were purified by column chromatography. The HR-MS was afforded from a mass spectrometer (Waters Xevo G2-XS-Qtof, Milford, MA). The ^1^H NMR and ^13^C NMR spectra of compounds were afforded on a Varian INOVA at 400 and 100 MHz, respectively.

#### The synthesis of compounds 3a and 3b was presented in supporting information

##### General preparation procedures of the target compounds 5a–5h

**3a** or **3b** (2.0 mmol) and K_2_CO_3_ (3.5 mmol) were added to CH_3_CN solution (10 mL). Under ice-water bath conditions, *N*,*N*-disubstituted carbamoyl chlorides (**4a**–**4e**) (3.2 mmol) were added to the reaction. The reaction solution was reacted for 15–18 h under reflux conditions. When the reaction was complete, the solvent was evaporated to dryness under reduced pressure. Water (30 mL) was added to the residue and extraction was carried out using CH_2_Cl_2_ (2 × 30 mL). The combined organic phases were washed with saturated sodium chloride (50 mL) and dried with anhydrous sodium sulphate. The solvent was dried under reduced pressure to afford the crude product, which was further purified by column chromatography using petroleum ether/ethyl acetate (50:1) as an eluent to gain the target compounds **5a**–**5h**.

###### 4-(4-Benzylpiperidine-1-carbonyl)-1,3-phenylene bis(dimethylcarbamate) (5a)

**3a** was reacted with dimethylcarbamoyl chloride (**4a**) according to the above general preparation to gain target derivative **5a**. Light yellow oil, 50.6% yield and 96.7% HPLC purity. ^1^H NMR (400 MHz, CDCl_3_) *δ* 7.28 (t, *J* = 7.2 Hz, 2H, 2 × Ar-H), 7.19 (t, *J* = 7.2 Hz, 2H, 2 × Ar-H), 7.13 (s, 1H, Ar-H), 7.11–7.09 (m, 2H, 2 × Ar-H), 7.02 (d, *J* = 7.2 Hz, 1H, Ar-H), 4.70 (d, *J* = 12.8 Hz, 1H, 1/2 PhCH_2_), 3.61 (d, *J* = 12.8 Hz, 1H, 1/2 PhCH_2_), 3.06 (s, 3H, NCH_3_), 3.02–2.93 (m, 10H, 3 × NCH_3_ + 1/2 NCH_2_), 2.88 (t, *J* = 13.2 Hz, 1H, 1/2 CH_2_), 2.66–2.63 (m, 1H, 1/2 NCH_2_), 2.54–2.52 (m, 2H, NCH_2_), 2.06–2.03 (m, 1H, CH), 1.75 (d, *J* = 10.8 Hz, 2H, CH_2_), 1.58 (d, *J* = 12.8 Hz, 1H, 1/2 CH_2_). ^13^C NMR (100 MHz, CDCl_3_) *δ* 166.4, 154.1, 152.2, 129.1, 128.3, 127.5, 126.4, 126.1, 118.5, 47.5, 43.0, 42.0, 38.6, 36.8, 36.7, 36.5, 32.5, 31.8. HR-ESI-MS: Calcd. for C_25_H_31_N_3_O_5_ [M + H]^+^: 454.2297, found: 454.2349.

###### 4-(4-Benzylpiperidine-1-carbonyl)-1,3-phenylene bis(ethyl(methyl)carbamate) (5b)

**3a** was reacted with *N*-ethyl-*N*-methylcarbamoyl chloride (**4b**) according to the above general preparation to gain target derivative **5b**. Light yellow oil, 43.8% yield and 96.5% HPLC purity. ^1^H NMR (400 MHz, CDCl_3_) *δ* 7.28 (t, *J* = 7.2 Hz, 2H, 2 × Ar-H), 7.19 (t, *J* = 7.2 Hz, 2H, 2 × Ar-H), 7.11 (d, *J* = 7.2 Hz, 3H, 3 × Ar-H), 7.03–7.00 (m, 1H, Ar-H), 4.70 (d, *J* = 12.8 Hz, 1H, 1/2 PhCH_2_), 3.61 (d, *J* = 12.8 Hz, 1H, 1/2 PhCH_2_), 3.46–3.34 (m, 5H, 2 × NCH_2_ + 1/2 NCH_2_), 3.03 (s, 3/2 H, 1/2 NCH_3_), 2.97 (s, 3H, NCH_3_), 2.95 (s, 3/2 H, 1/2 NCH_3_), 2.87 (t, *J* = 12.8 Hz, 1H, 1/2 NCH_2_), 2.65–2.61 (m, 1H, 1/2 CH_2_), 2.54–2.50 (m, 2H, NCH_2_), 1.96–1.94 (m, 1H, CH), 1.74 (d, *J* = 11.6 Hz, 2H, CH_2_), 1.57 (d, *J* = 12.8 Hz, 1H, 1/2 CH_2_), 1.21–1.14 (m, 6H, 2 × CH_3_). ^13^C NMR (100 MHz, CDCl_3_) *δ* 166.4, 153.8, 153.2, 152.2, 148.2, 139.9, 129.1, 128.3, 127.5, 126.4, 126.1, 118.6, 116.9, 116.7, 116.6, 53.5, 47.5, 44.2, 43.0, 42.0, 38.3, 38.2, 34.3, 33.9, 33.8, 32.6, 31.7, 13.2, 12.6, 12.4. HR-ESI-MS: Calcd. for C_27_H_35_N_3_O_5_ [M + H]^+^: 482.2610, found: 482.2648.

###### 4-(4-Benzylpiperidine-1-carbonyl)-1,3-phenylene bis(diisopropylcarbamate) (5c)

**3a** was reacted with diisopropylcarbamoyl chloride (**4c**) according to the above general preparation to gain target derivative **5c**. Light yellow oil, 32.9% yield and 96.6% HPLC purity. ^1^H NMR (400 MHz, CDCl_3_) *δ* 7.27 (t, *J* = 7.6 Hz, 2H, 2 × Ar-H), 7.19 (t, *J* = 7.2 Hz, 2H, 2 × Ar-H), 7.15–7.08 (m, 5H, 5 × Ar-H), 4.68 (d, *J* = 12.8 Hz, 1H, 1/2 PhCH_2_), 4.14–3.83 (m, 5H, 1/2 PhCH_2_ + 4 × NCH), 3.70 (t, *J* = 12.8 Hz, 1H, 1/2 NCH_2_), 2.97–2.82 (m, 1H, 1/2 NCH_2_), 2.71–2.58 (m, 1H, 1/2 CH_2_), 2.53–2.41 (m, 2H, NCH_2_), 1.87–1.82 (m, 1H, CH), 1.72 (d, *J* = 10.8 Hz, 2H, CH_2_), 1.60 (d, *J* = 11.6 Hz, 1H, 1/2 CH_2_), 1.33 (d, *J* = 6.4 Hz, 12H, 4 × CH_3_), 1.24 (d, *J* = 6.4 Hz, 12H, 4 × CH_3_). ^13^C NMR (100 MHz, CDCl_3_) *δ* 166.0, 153.4, 140.0, 129.7, 129.0, 128.3, 126.0, 122.9, 122.8, 120.3, 47.5, 46.7, 46.6, 43.1, 43.0, 42.0, 38.4, 31.6, 21.7, 21.6, 21.5, 21.3, 20.7, 20.6, 20.5. HR-ESI-MS: Calcd. for C_33_H_47_N_3_O_5_ [M + H]^+^: 566.3549, found: 566.3602.

###### 4-(4-Benzylpiperidine-1-carbonyl)-1,3-phenylene bis(pyrrolidine-1-carboxylate) (5d)

**3a** was reacted with 1-pyrrolidinecarbonylchloride (**4d**) according to the above general preparation to gain target derivative **5d**. Light yellow oil, 33.2% yield and 96.5% HPLC purity. ^1^H NMR (400 MHz, CDCl_3_) *δ* 7.28 (t, *J* = 7.2 Hz, 2H, 2 × Ar-H), 7.19 (t, *J* = 6.4 Hz, 2H, 2 × Ar-H), 7.11 (d, *J* = 7.2 Hz, 3H, 3 × Ar-H), 7.04 (d, *J* = 8.0 Hz, 1H, Ar-H), 4.70 (d, *J* = 12.8 Hz, 1H, 1/2 PhCH_2_), 3.60 (d, *J* = 12.8 Hz, 1H, 1/2 PhCH_2_), 3.53 (t, *J* = 6.8 Hz, 2H, NCH_2_), 3.46 (t, *J* = 6.8 Hz, 2H, NCH_2_), 3.43–3.40 (m, 1H, 1/2 NCH_2_), 3.38 (t, *J* = 6.8 Hz, 4H, 2 × NCH_2_), 2.92–2.83 (m, 1H, 1/2 NCH_2_), 2.68–2.62 (m, 1H, 1/2 CH_2_), 2.55–2.52 (m, 2H, NCH_2_), 1.97–1.85 (m, 9H, 4 × CH_2_ + CH), 1.76–1.74 (m, 2H, CH_2_), 1.58 (d, *J* = 12.8 Hz, 1H, 1/2 CH_2_). ^13^C NMR (100 MHz, CDCl_3_) *δ* 166.6, 152.4, 148.6, 139.9, 129.1, 128.3, 126.1, 118.4, 112.8, 48.0, 46.5, 46.4, 25.8, 25.5, 24.9. HR-ESI-MS: Calcd. for C_29_H_35_N_3_O_5_ [M + H]^+^: 506.2610, found: 506.2641.

###### 4-(4-Benzylpiperidine-1-carbonyl)-1,3-phenylene bis(morpholine-4-carboxylate) (5e)

**3a** was reacted with 4-morpholinecarbonyl chloride (**4e**) according to the above general preparation to gain target derivative **5e**. Light yellow oil, 28.6% yield and 96.6% HPLC purity. ^1^H NMR (400 MHz, CDCl_3_) *δ* 7.28 (t, *J* = 7.2 Hz, 2H, 2 × Ar-H), 7.20 (t, *J* = 7.6 Hz, 2H, 2 × Ar-H), 7.12 (d, *J* = 6.4 Hz, 3H, 3 × Ar-H), 7.03 (d, *J* = 8.4 Hz, 1H, Ar-H), 4.70 (d, *J* = 12.8 Hz, 1H, 1/2 PhCH_2_), 3.73–3.50 (m, 17H, 4 × OCH_2_ + 1/2 PhCH_2_ + 4 × NCH_2_), 3.27 (t, *J* = 4.8 Hz, 1H, 1/2 NCH_2_), 2.88–2.86 (m, 1H, 1/2 CH_2_), 2.67–2.59 (m, 1H, 1/2 NCH_2_), 2.55 (d, *J* = 6.0 Hz, 2H, NCH_2_), 1.91–1.88 (m, 1H, CH), 1.77 (d, *J* = 11.2 Hz, 2H, CH_2_), 1.59 (d, *J* = 12.8 Hz, 1H, 1/2 CH_2_). ^13^C NMR (100 MHz, CDCl_3_) *δ* 166.1, 152.9, 152.5, 151.9, 139.7, 129.1, 128.3, 127.7, 126.6, 126.1, 118.7, 116.6, 66.6, 53.5, 47.6, 47.2, 45.0, 44.9, 44.2, 43.0, 42.1, 32.6, 31.8. HR-ESI-MS: Calcd. for C_29_H_35_N_3_O_7_ [M + H]^+^: 538.2509, found: 538.2565.

###### 2-(4-Benzylpiperidine-1-carbonyl)-1,4-phenylene bis(dimethylcarbamate) (5f)

**3b** was reacted with dimethylcarbamoyl chloride (**4a**) according to the above general preparation to gain target derivative **5f**. Light yellow oil, 34.3% yield and 96.8% HPLC purity. ^1^H NMR (400 MHz, CDCl_3_) *δ* 7.27 (t, *J* = 7.2 Hz, 2H, 2 × Ar-H), 7.20 (t, *J* = 8.8 Hz, 2H, 2 × Ar-H), 7.13–7.10 (m, 4H, 4 × Ar-H), 4.67 (d, *J* = 11.6 Hz, 1H, 1/2 PhCH_2_), 3.65 (d, *J* = 13.6 Hz, 1H, 1/2 PhCH_2_), 3.07–2.96 (m, 13H, 4 × NCH_3_ + 1/2 NCH_2_), 2.91–2.87 (m, 1H, 1/2 NCH_2_), 2.64 (dt, *J*_1_ = 10.8 Hz, *J*_2_ = 2.0 Hz, 1H, 1/2 CH_2_), 2.54–2.52 (m, 2H, NCH_2_), 1.85–1.82 (m, 1H, CH), 1.75 (d, *J* = 10.8 Hz, 2H, CH_2_), 1.60 (d, *J* = 11.6 Hz, 1H, 1/2 CH_2_). ^13^C NMR (100 MHz, CDCl_3_) *δ* 165.8, 154.4, 154.1, 148.3, 139.9, 130.2, 129.1, 128.3, 126.1, 123.7, 122.9, 120.4, 53.5, 47.5, 43.1, 43.0, 42.0, 38.3, 38.2, 36.8, 36.5, 32.4, 31.7. HR-ESI-MS: Calcd. for C_25_H_31_N_3_O_5_ [M + H]^+^: 454.2297, found: 454.2330.

###### 2-(4-Benzylpiperidine-1-carbonyl)-1,4-phenylene bis(ethyl(methyl)carbamate) (5g)

**3b** was reacted with *N*-ethyl-*N*-methylcarbamoyl chloride (**4b**) according to the above general preparation to gain target derivative **5g**. Light yellow oil, 39.7% yield and 97.1% HPLC purity. ^1^H NMR (400 MHz, CDCl_3_) *δ* 7.27 (t, *J* = 7.2 Hz, 2H, 2 × Ar-H), 7.23–7.17 (m, 2H, 2 × Ar-H), 7.14–7.18 (m, 4H, 4 × Ar-H), 4.68 (d, *J* = 12.8 Hz, 1H, 1/2 PhCH_2_), 3.65 (d, *J* = 13.2 Hz, 1H, 1/2 PhCH_2_), 3.45–3.38 (m, 5H, 2 × NCH_2_ + 1/2 NCH_2_), 3.04 (s, 3/2 H, 1/2 NCH_3_), 2.97 (s, 3H, NCH_3_), 2.94 (s, 3/2 H, 1/2 NCH_3_), 2.88 (t, *J* = 12.8 Hz, 1H, 1/2 NCH_2_), 2.65–2.59 (m, 1H, 1/2 CH_2_), 2.55–2.49 (m, 2H, NCH_2_), 1.76–1.69 (m, 3H, CH + CH_2_), 1.59 (d, *J* = 12.4 Hz, 1H, 1/2 CH_2_), 1.24–1.09 (m, 6H, 2 × CH_3_). ^13^C NMR (100 MHz, CDCl_3_) *δ* 165.8, 153.9, 153.6, 148.4, 148.3, 139.9, 130.2, 129.1, 128.5, 128.3, 126.1, 123.7, 122.9, 122.8, 120.4, 47.5, 47.4, 44.1, 43.1, 43.0, 41.9, 34.3, 33.9, 32.5, 31.7, 13.2, 12.5. HR-ESI-MS: Calcd. for C_27_H_35_N_3_O_5_ [M + H]^+^: 482.2610, found: 482.2702.

###### 2-(4-Benzylpiperidine-1-carbonyl)-1,4-phenylene bis(diisopropylcarbamate) (5h)

**3b** was reacted with diisopropylcarbamoyl chloride (**4c**) according to the above general preparation to gain target derivative **5h**. Light yellow oil, 23.6% yield and 96.5% HPLC purity. ^1^H NMR (400 MHz, CDCl_3_) *δ* 7.27 (t, *J* = 7.6 Hz, 2H, 2 × Ar-H), 7.23–7.15 (m, 2H, 2 × Ar-H), 7.13–7.08 (m, 2H, 2 × Ar-H), 7.07–7.05 (m, 1H, Ar-H), 7.03–6.98 (m, 1H, Ar-H), 4.68 (d, *J* = 12.0 Hz, 1H, 1/2 PhCH_2_), 4.06–3.88 (m, 5H, 1/2 PhCH_2_ + 4 × NCH), 3.65–3.61 (d, *J* = 8.4 Hz, 1H, 1/2 NCH_2_), 2.93–2.80 (m, 1H, 1/2 NCH_2_), 2.59–2.56 (m, 1H, 1/2 CH_2_), 2.54–2.51 (m, 2H, NCH_2_), 1.85–1.79 (m, 1H, CH), 1.73 (d, *J* = 10.0 Hz, 2H, CH_2_), 1.58 (d, *J* = 13.2 Hz, 1H, 1/2 CH_2_), 1.30–1.24 (m, 24H, 8 × CH_3_). ^13^C NMR (100 MHz, CDCl_3_) *δ* 166.5, 153.0, 152.0, 139.9, 129.1, 128.4, 127.3, 126.6, 126.1, 118.7, 117.0, 112.2, 111.0, 47.6, 46.9, 46.2, 43.1, 38.3, 32.7, 32.3, 31.7, 21.6, 21.3, 20.7, 20.5. HR-ESI-MS: Calcd. for C_33_H_47_N_3_O_5_ [M + H]^+^: 566.3549, found: 566.3588.

## Supplementary Material

Supplemental Material
