# Supplementary material for: Development of novel salicylic acid–donepezil–rivastigmine hybrids as multifunctional agents for the treatment of Alzheimer’s disease
Source: J Enzyme Inhib Med Chem. 2023 Jul 6;38(1):2231661. doi: 10.1080/14756366.2023.2231661 (PMC10327518; doi:10.1080/14756366.2023.2231661)
Supplement: Supplemental Material [file IENZ_A_2231661_SM7422.pdf]

Supporting information

Development of novel salicylic acid-donepezil-rivastigmine hybrids as multifunctional agents for the treatment of Alzheimer's disease

Yi Zhou[1,#], Ying He[1,#], Teng Xue[2,#], Jing Mi[1], Jing Yang[1], Rongrui Wei[3], Wenmin Liu[1,*], Qinge Ma[3,*], Zhenghuai Tan[4,*], Zhipei Sang[1,2,*].

[1]College of Chemistry and Pharmaceutical Engineering, Nanyang Normal University, Nanyang, 473061, China

[2]Key Laboratory of Tropical Biological Resources of Ministry of Education and One Health Institute, School of Pharmaceutical Sciences, Hainan University, Haikou, 570228, China

[3] Key Laboratory of Modern Preparation of Traditional Chinese Medicine of Ministry of Education, Research Center of Natural Resources of Chinese Medicinal Materials and Ethnic Medicine, Jiangxi University of Traditional Chinese Medicine, Nanchang, 330004, China

[4]Institute of Traditional Chinese Medicine Pharmacology and Toxicology, Sichuan Academy of Chinese Medicine Sciences, Chengdu, 610041, China

**Corresponding Author*.

E-mail:sangzhipei@126.com (Zhipei Sang)

E-mail: tanzhh616@163.com (Zhenghuai Tan)

E-mail:liuwm1969@163.com (Wenmin Liu)

E-mail: maqinge2006@163.com (Qinge Ma)

#These authors contributed equally.

Contents

1. Synthesis of compounds 3a and 3b

2. Biological activity experiments

3. Table S1 Prediction of drug-likeness properties of compound 5a.

4. Representative ^{1}H, ^{13}C NMR and HR-ESI-MS spectra

1. Synthesis of compounds 3a and 3b

General preparation procedures of compounds **3a** and **3b**. The starting material **1a** or **1b** (2,4-dihydroxybenzoic acid **1a**; 2,5-dihydroxybenzoic acid **1b**) (2 mmol) was dissolved in 10 mL THF. Then, EDCI (3.0 mmol), HOBT (3.0 mmol) and excessive amounts (2.2 mmol) of 4-benzylpiperidine (**2**) were added into the solution, respectively. The reaction mixture was stirred at room temperature overnight and was monitored by TLC. After the reaction completed, the solvent was evaporated under reduced pressure. The crude residue was dissolved in 30 mL water, washed with CH_2Cl_2 (2 × 30 mL), the combined organic solvent was washed with saturated NaCl (80 mL) and dried with Na_2SO_4. The organic solvent was evaporated under reduced pressure to obtain crude residue, which was further purified by silica gel chromatography (petroleum ether/acetone = 30: 1) to obtain the key intermediate compounds **3a** and **3b**.

(4-Benzylpiperidin-1-yl)(2,4-dihydroxyphenyl)methanone (**3a**). Light yellow oil, 50.1% yield, and 97.1% HPLC purity. ^{1}H NMR (400 MHz, $CDCl_3$) δ 10.12 (s, 1H, OH), 7.32-7.25 (m, 3H, 3 × Ar-H), 7.21 (t, *J* = 7.2 Hz, 1H, Ar-H), 7.14 (d, *J* = 7.2 Hz, 2H, 2 × Ar-H), 7.09 (d, *J* = 8.4 Hz, 1H, Ar-H), 6.42 (s, 1H, OH), 6.32 (d, *J* = 8.4 Hz, 1H, Ar-H), 4.32 (d, *J* = 12.8 Hz, 2H, phCH_2), 2.89 (t, *J* = 8.4 Hz, 2H, NCH_2), 2.57 (d, *J* = 7.2 Hz, 2H, NCH_2), 1.84-1.71 (m, 1H, CH), 1.73 (d, J = 12.8 Hz, 2H, CH_2), 1.32-1.12 (m, 2H, CH_2). ^{13}C NMR (100 MHz, $CDCl_3$) δ 171.2, 160.2, 160.1, 139.9, 130.0, 129.1 (2C), 128.4 (2C), 126.1, 110.1, 107.1, 104.2, 46.4 (2C), 42.9, 38.3, 32.2 (2C). HR-ESI-MS: Calcd. for $C_{19}H_{21}NO_3$ $[M+H]^+$: 312.1555, found: 312.1599.

(4-Benzylpiperidin-1-yl)(2,5-dihydroxyphenyl)methanone (**3b**). Light yellow oil, 50.1% yield, and 97.1% HPLC purity. ^{1}H NMR (400 MHz, $CDCl_3$) δ 8.50-8.48 (brs, 1H, OH), 7.28 (t, *J* = 7.2 Hz, 2H, 2 × Ar-H), 7.20 (t, *J* = 7.2 Hz, 1H, Ar-H), 7.13 (d, *J* = 7.6 Hz, 2H, 2 × Ar-H), 6.76 (s, 1H, Ar-H), 6.67 (d, *J* = 2.2 Hz, Ar-H), 4.31 (d, *J* = 12.4 Hz, 2H, phCH_2), 2.86 (t, *J* = 12.4 Hz, 2H, NCH_2), 2.55 (d, *J* = 7.2 Hz, 2H, NCH_2), 2.05 (s, 1H, CH), 1.82-1.76 (m, 2H, CH_2), 1.71 (d, *J* = 13.6 Hz, 2H, CH_2). ^{13}C NMR (100 MHz, $CDCl_3$) δ 170.0, 151.0, 148.0, 139.8, 129.0, 128.3, 126.1, 119.7, 118.5, 114.3, 42.8, 38.2, 32.2, 21.0, 14.2. HR-ESI-MS: Calcd. for $C_{19}H_{21}NO_3$ $[M+H]^+$:

312.1555, found: 312.1602.

2 Biological activity

2.1 Inhibition experiments of AChE and BuChE. The modified Ellman method was performed to test the AChE and BChE inhibitory ability of the compounds, using *ee*AChE and eqBChE (Sigma-Aldrich Co.). The detailed procedure referenced our previous work.[1,2]

2.2 Anti-inflammatory property. BV-2 cells were cultured in DMEM containing 10% fetal bovine serum, 100 U/mL penicillin, 100 U/mL streptomycin, and 1% nonessential amino acid at 37°C with 5% CO_2.

BV-2 cells in logarithmic growth stage were digested and centrifuged, and then resuspended in DMEM medium containing 1% penicillin-streptomycin and 10% fetal bovine serum. The cells was adjusted to 1.2×10^4 cells/well, and then seeded evenly in 96-well cell culture plates. The cells were incubated for 36 h at 37°C with 5% CO_2. After incubation, the cells were added to different concentrations (10 and 2 μM) of tested compounds, and incubated for 24h at 37°C with 5% CO_2. Then, 10 μL CCK-8 solution was added to each well, and incubated for another 1h at 37°C with 5% CO_2. The OD values were tested at 450 nM. The cell viability (%) $=\{(OD_{drug}-OD_{blank})\} /\{(OD_{control}-OD_{blank})\} \times 100\%$.

BV-2 cells (2×10^5 cells/well) were seeded in a 24-well cell culture plates. The cells were divided into blank control group, model group (1μg/mL LPS), LPS + 2 μM compound group, LPS + 1 μM compound group. Each drug group was pretreated with conrresponding concentration of drug solution for 8h. After 8h, the culture medium was aspirated out. Except for the blank control group, which was added with DMEM medium, the other groups were stimulated with 1 μg/mL LPS for 24h. Then cell supernatant was aspirated and centrifuged at 3000 rpm for 3 min. The content of NO in cell culture supernatant was determined by Griess method, and the levels of cytokines IL-6 and IL-1*β* in cell culture supernatant were determined by IL-6 ELISA kit and IL-1*β* ELISA kit, respectively.

2.3 Neuroprotective effects on Aβ$_{25-35}$-induced PC12 cells injury.

(1) Materials and reagents. PC12 cell (Purchased from the Cell Bank of Shanghai Institute of Cell Biology, Chinese Academy of Sciences), DME H-21 4.5g/Liter Glucose(Gibco), Fetal bovine serum (Vicente), cellcountingkit-8, CCK8(Shanghai Biyuntian Biotechnology Co., Ltd), 1% penicillin-streptomycin (Double antibody, cellmax), Double antibody (Beijing Solebao Technology Co., Ltd), 0.25% trypsin solution (Beijing Solebao Technology Co., Ltd), a phosphate-buffered solution, (PBS, Beijing Solebao Technology Co., Ltd), DMSO(Tianjin Kemio Chemical Reagent Company), Aβ_{25-35} (Purity>98% , Sigma Corporation, USA), The experimental water is double distilled water; Other reagents are analytical pure.

(2) Istrument. Cell culture incubator(Thermo Scientific), Ultra-clean workbench(Jiangsu Sujing Group Co., Ltd), Inverted phase-contrast fluorescence microscope(Japan Nikon Corporation), Varioska full-wavelength multifunction microplate reader(Thermo Scientific), Digital display constant temperature water bath pot(Bangsi Instrument Technology Co., Ltd), 1/100,000 electronic balance(Shanghai Jinghai Instrument Co., Ltd).

In this experiment, the cell viability was detected using CCK-8 kit and microplate reader.

(3) PC12 Cell complete medium formulation. Recover PC12 cells, use 10% fetal bovine serum 50mL, 5% equine serum 25mL and 1% penicillin mixture 5mL, and finally add DMEM high-sugar medium solution to 500mL, that is, to obtain.

(4) PC12 Cell Recover. Quickly remove the cells from the -80 °C freezer, shake in a 37 °C water bath pot to dissolve quickly, disinfect the cryopreservation tube and transfer it to the ultra-clean table, transfer the cryopreservation in the cryopreservation tube to a 15 mL centrifuge tube, add 3 mL of complete medium, gently blow well with a pipette, centrifuge at 1000 *g at room temperature for 5 min, discard the upper layer of medium, add 4 mL complete medium again, gently blow well and transfer to the culture flask, transfer to the incubator at 37 °C, 5% CO2, saturation humidity, and change the liquid every other day.

(5) PC12 Cell Passage. During the logarithmic growth phase of cell growth, add 2 mL PBS washing, digest with 1 mL 0.25% pancreatic enzyme for 2 min, add 2 mL complete medium to stop digestion, transfer to a 15 mL centrifuge tube, centrifuge at 1000 *g at room temperature for 5 min, discard the upper layer medium, 1:4 subculture.

(6) PC12 Cell Cryopreservation. Take logarithmic growth stage cells, digest according to normal passage, add 1 mL of cryoster, resuspend the cells, and store at 4°C for 30 min. Freeze at -20 °C for 2 h and then transfer to -80 °C for storage. Try to freeze cells with advanced algebraic numbers for subsequent experiments.

(7) Preparation of sample solutions. Precisely weigh the appropriate amount of sample, DMSO is formulated into 100 mmol/L mother slurry, stored at -20 °C for backup, and diluted with complete medium for final concentration of 5, 10, 20 μmol/L of drug solution when used, so that the final DMSO concentration into the cell is 0.05%, avoiding toxicity to the cell.

(8) $A\beta_{25\text{-}35}$ of aggregation. 1 mg $A\beta_{25\text{-}35}$ dissolved in 943 μL of sterilized deionized water, formulated into 1 mmol/L of mother liquor, aliquoted, –20 °C preservation. Remove the appropriate volume in a 48-well plate and incubate in a 37 °C incubator for 2 days to allow its aging to accumulate and produce neurotoxicity. After aggregation is complete, place at -20 °C for standby and dilute with PBS before use so that the final concentration of its entry into the cells is 25 μmol/L. Seal with plastic wrap and band during culture to prevent loss of moisture evaporation.

(9) Neuroprotective effect of the compound on the injury of PC12 cells after $A\beta_{25\text{-}35}$ induction. The PC12 cells during the logarithmic growth phase are digested and centrifuged with pancreatic enzyme solution, the cells are resuspended by adding the prepared media solution, and piped evenly with a pipette to seed in a 96-well cell culture plate (cell concentration adjusted to 2.0×10^{4} cell·mL^{-1}). Add 100 μL of complete medium per well and incubate at 37 °C under 5% CO_2 conditions for 24h.

After 24 h, PC12 cells were divided into blank group, control group, $A\beta_{25\text{-}35}$ model group and experimental group. The control group and the model group did not add drugs, discarded the original medium, and added a new complete medium of 100

μL, and the experimental group added 100 μL of complete medium containing different concentrations of drugs, so that the final concentrations of drugs were 5, 10, 20 μmol/L, 37 °C, 5% CO_2 conditions for 2h.

After 2 h, the dosing group and the model group were added to $A\beta_{25-35}$ solution (so that the final concentration of $A\beta_{25-35}$ after addition was 25 μmol/L) 10 μL, after incubation at 37 °C, 5% CO_2 conditions for 24 h, 10 μL CCK-8 per well was added at 37 °C and 5% CO_2 to continue the incubation, after 1h, the OD value was measured at a wavelength of 450 nm using a microplate reader, and 3 complex wells were set for each concentration, and the cell survival rate was determined according to the following formula:

Cell viability(%) = $(A_{sample}-A_{blank})/(A_{control}-A_{blank}) \times 100\%$. A_{sample}, A_{blank} and $A_{control}$ represent UV absorption at 450 nm for cells treated by therapeutic agents, culture medium, or vehicle, respectively.

2.4 Stability assays.[3]

(1) The artificial gastrointestinal fluids were prepared on the basis of the standard method described in China Pharmacopoeia (version 2015). The artificial gastric fluid consisted of HCl (0.045 mol/L) and pepsin (10 g/L), while the artificial intestinal fluid consisted of trypsin (10 g/L) and KH_2PO_4 (6.8 g/L), and the pH was adjusted to 6.8 with 0.1 mol/L NaOH. The blank gastric fluid and blank intestinal fluid was similar as described above without pepsin and trypsin. Compound **5a** (1 mg/mL, n = 3) was incubated with artificial gastric fluid, blank gastric fluid, artificial intestinal fluid and blank intestinal fluid at 37 ◦C, and 600 μL methanol was then added into the reagent to stop reaction at time of 0, 0.5, 1, 2, 3, 4 and 8 h. Supernatant was collected by centrifugation at 13,000 rpm for 10 min and analyzed by UPLC-MS/MS. The data were processed by GraphPad Prism 8.0 software.

(2) The stability of compound **5a** was performed in rat liver microsomes. The rat liver microsomes was purchased from Dalian Meilun Biotechnology Co., Ltd, DaLian, China. Briefly, solution A (10 mL) contained G-6-P-Na_2 (200 mg), $NADPNa_2$ (200 mg), $MgCl_2$ (133 mg), and H_2O was used as a solvent. Solution B (25 mL) contained G-6-P-DH (1000 U), Na-Citrate2 (200 mg), and H_2O was used as a solvent. NADPH

solution (1 mM) was composed by solution A and solution B (*v/v* = 5:1). Compound was dissolved in DMSO to obtain 1 mg/mL stock solution. Rat liver microsomes was added to compound solution (the final concentration of compound and rat liver microsomes was 10 μg/mL and 0.5 mg/mL, respectively) (n = 3). The reaction was started by the addition of NADPH. The total mixture (200 μL) was incubated at 37 °C. Cold methanol (600 μL) was added at 0, 5, 15, 20, 30, 45 and 60 min, respectively, to stop the reaction. Samples were centrifuged at 13000 rpm for 10 min at 4 °C, and 100 μL supernatant was removed and analyzed by UPLC-MS/MS. The stability results were presented as % remaining *vs.* time. The experiments were conducted in triplicate.

(3) The stability of compound was performed in rat plasma. Briefly, compound (1 mg/mL) were dissolved in DMSO, and the reaction (10 μg/mL, n = 3) was prepared by mixing 198 μL plasma with 2 μL compounds solution, then incubated in 37 ◦C and stopped by adding 600 μL methanol at time of 0, 0.5, 1, 1.5, 2 and 3 h, followed by centrifugation at 13,000 rpm for 10 min at 4 °C. The supernatant was dried and redissolved in 100 μL methanol. After centrifugation, the supernatant was analyzed by UPLC-MS/MS. The stability data was presented as remaining percentage vs time.

2.5 In vivo assay

Acute toxicity. Kunming mice at body weight of 18–22 g (six weeks old, either gender) were supplied by the Center of Experimental Animals of Sichuan Academy of Chinese Medicine Sciences (eligibility certification no. SCXK-Chuan2018-19). Mice were maintained under standard conditions with a 12 h:12 h light–dark cycle at 20−22 °C with a relative humidity of 60−70%. Sterile food and water were provided according to institutional guidelines. Prior to each experiment, mice were fasted overnight and allowed free access to water. Compound **5a** at doses of 250, 500 and 1000 mg/kg (n = 6 per group) by intragastric administration. After the administration of the compound **5a**, the mice were observed continuously for the first 4 h for any abnormal behavior and mortality changes, intermittently for the next 24 h, and occasionally thereafter for 14 days for the onset of any delayed effects. All animals were sacrificed on the 14th day after drug administration and were macroscopically

examined for possible damage to the heart, liver, and kidneys.

Assay method. The step-down passive avoidance task was employed to investigate the effects of **5a** on scopolamine-induced memory impairment.[21,22] Sixty mice were random divide into six groups. They were compound **5a** group (9.0 mg/kg, 18.0 mg/kg and 36.0 mg/kg), same volume of water (untreated group), model group (3 mg/kg scopolamine), 10.0 mg/kg rivastigmine. After 30 min, memory impairment was induced by administering scopolamine (3 mg/kg). Then 30 min later, the learning and memory capacity of mouse were measured by the Y-maze test. The maze was made of black-colored acryl and positioned at equal angles. Rats were placed at the end of the arm and allowed to move freely through the maze during 8 min sessions. Arm entry sessions were recorded when the hind paws of the rat were completely placed in the arm. Consecutive entry into three arms in alternative order was defined as successive entries on overlapping triplet sets and the alternation percentage was calculated as the ratio of actual to possible alternations (defined as the total number of arm entries minus 2), multiplied by 100.

References

(1) Sang Z, Wang K, Han X, Cao M, Tan Z, Liu W. Design, synthesis, and evaluation of novel ferulic acid derivatives as multi-target-directed ligands for the treatment of Alzheimer's disease. *ACS Chem Neurosci*. **2019**, 10(2): 1008-1024.

(2) Sang Z, Qiang X, Li Y, Yuan W, Liu Q, Shi Y, Deng Y. Design, synthesis and evaluation of scutellarein-O-alkylamines as multifunctional agents for the treatment of Alzheimer's disease. Eur J Med Chem, 2015, 94, 348-366.

(3) Sang Z, Bai P, Ban Y, Wang K, Wu A, Mi J, Hu J, Xu R, Zhu G, Wang J, Zhang J, Wang C, Tan Z, Tang L. Novel donepezil-chalcone-rivastigmine hybrids as potential multifunctional anti-Alzheimer's agents: Design, synthesis, in vitro biological evaluation, in vivo and in silico studies. Bioorg Chem. 2022, 127: 106007.

3.**Table S1** Prediction of drug-likeness properties of compound **5a**.

**Com.**	**MW**	**DHB**	**AHB**	**QPlogPw**	**QPlog Po/w**	**QPlogS**	**PSA**	**QPlog HERG**	**QPP Caco**
**5a**	453.537	0	9	11.754	3.799	-5.488	96.149	-6.082	844.584
**Com.**	**QPP MDCK**	**QPlogKhsa**	**QPlogBB**	**#Metab**	**%HOA**	**CNS**	**RoF**	**RoT**	
**5a**	412.154	0.175	-0.944	1	100	-1	0	0	

MW: Molecular weight (< 500).

DHB: Number of hydrogen bond donors (< 5).

AHB: Number of hydrogen bond acceptors (< 10).

QPlogPw: Water/gas partition coefficient.

QPlogPo/w: Octanol/water partition coefficient (< 5).

QPlogS: Aqueous solubility (-6.5 to 0.5).

PSA: Polar surface area (7–200).

QPlog HERG: IC50 value for blockage of HERG Kþ channels (< -5).

QPP Caco: apparent Caco-2 permeability (nm/sec) (< 25 poor, > 500 great).

QPP MDCK: apparent MDCK permeability (nm/sec) (< 25 poor, > 500 great).

QPlog Khsa: Prediction of binding to human serum albumin (-1.5 to 1.5).

QPlogBB: Predicted brain/blood partition coefficient (-3.0 to 1.2).

%HOA: Percent Human Oral Absorption (> 80% is high < 25% is poor).

CNS: Predicted central nervous system activity (-2.0 to + 2.0).

ROF: rule of fve.

ROT: rule of three.

3.Representative ^{1}H, ^{13}C NMR and HR-ESI-MS spectra

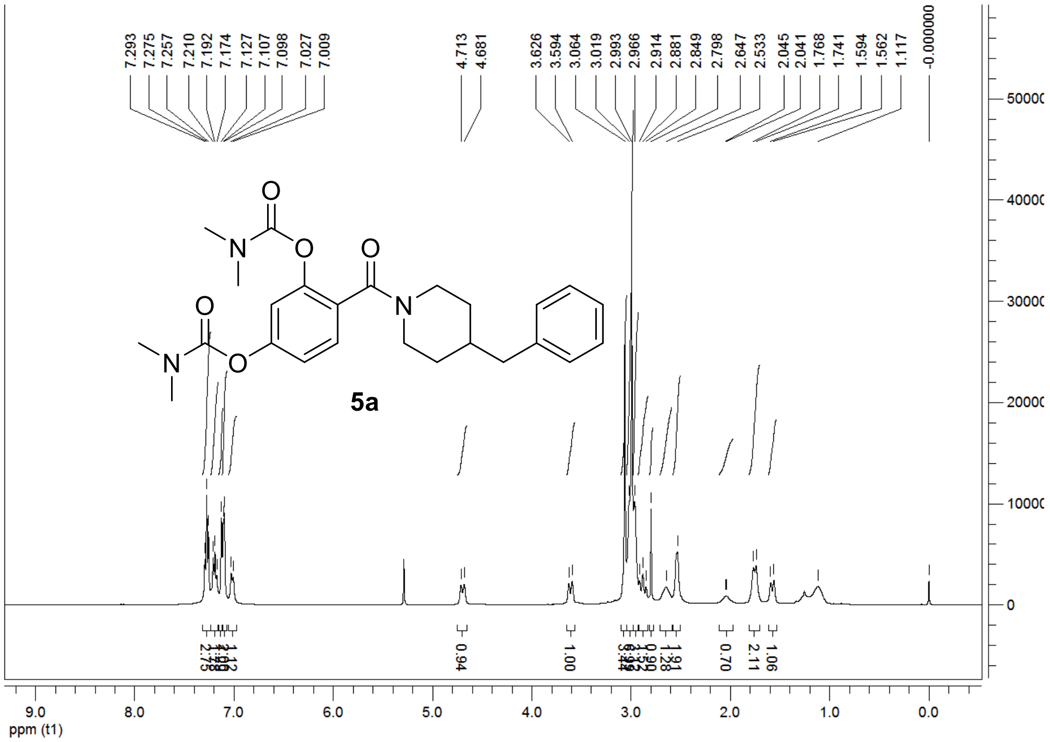
7.293 7.275 7.257 7.210 7.192 7.174 7.127 7.107 7.098 7.027 7.009
4.713 4.681
3.626 3.594 3.064 3.019 2.993 2.966 2.914 2.881 2.849 2.798 2.647 2.533 2.045 2.041 1.768 1.741 1.594 1.562 1.117
-0.000000
5a
2.75 2.12 0.94 1.00 1.28 1.91 0.70 2.11 1.06
9.0 8.0 7.0 6.0 5.0 4.0 3.0 2.0 1.0 0.0
ppm (t1)
50000 40000 30000 20000 10000 0

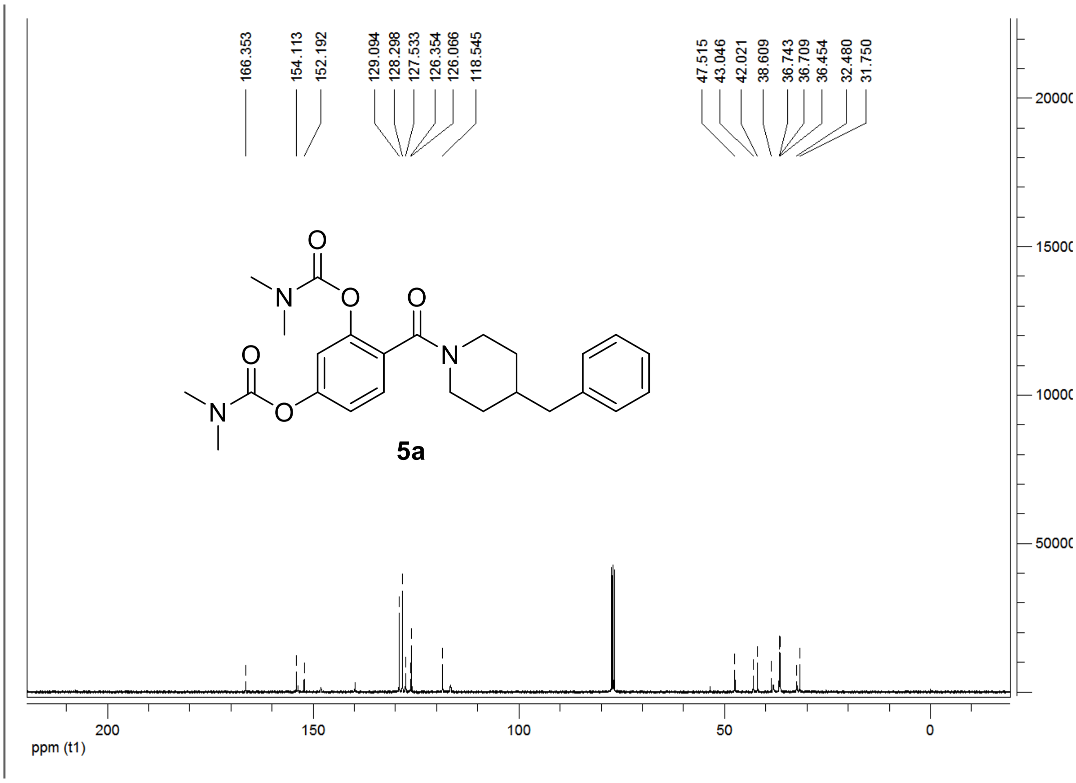
166.353
154.113 152.192
129.094 128.298 127.533 126.354 126.066 118.545
47.515 43.046 42.021 38.609 36.743 36.709 36.454 32.480 31.750
5a
200 150 100 50 0
ppm (t1)
20000 15000 10000 50000

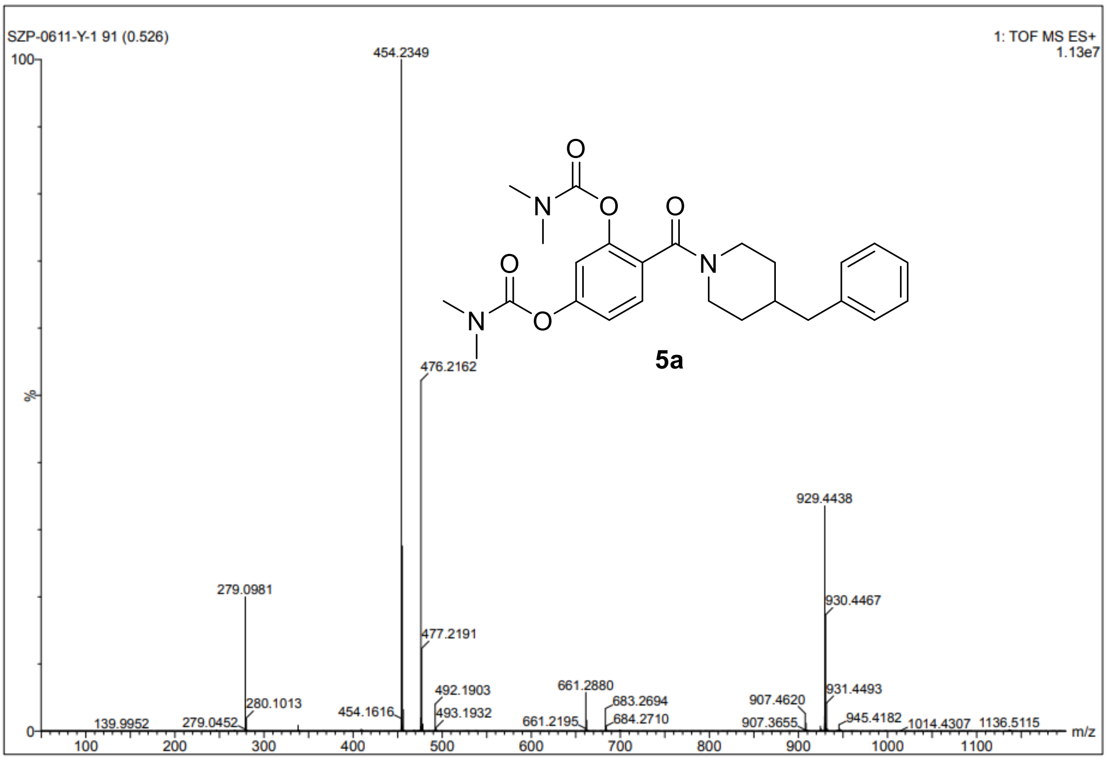
SZP-0611-Y-1 91 (0.526)
1: TOF MS ES+
1.13e7
454.2349
476.2162
929.4438
279.0981
930.4467
477.2191
492.1903
493.1932
661.2880
683.2694
684.2710
907.4620
931.4493
280.1013
454.1616
139.9952
279.0452
661.2195
907.3655
945.4182
1014.4307
1136.5115
m/z
%
5a

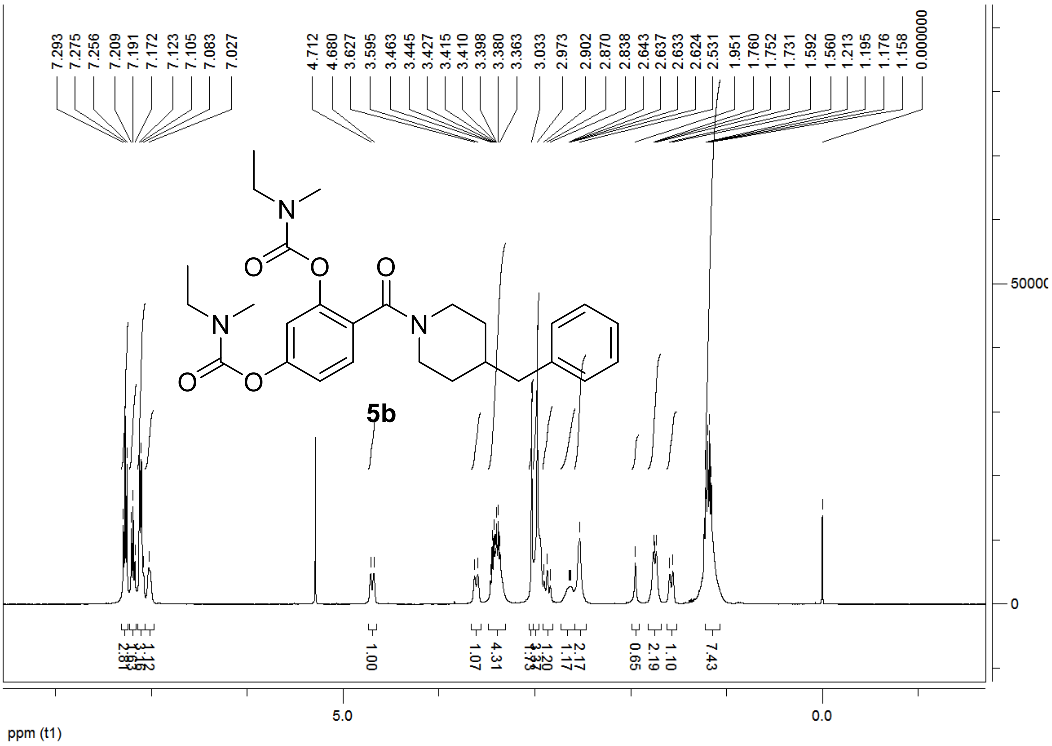
5b
ppm (t1)
5.0
0.0
50000
7.43
1.00
1.07
4.31
2.17
1.17
0.65
2.19
1.10

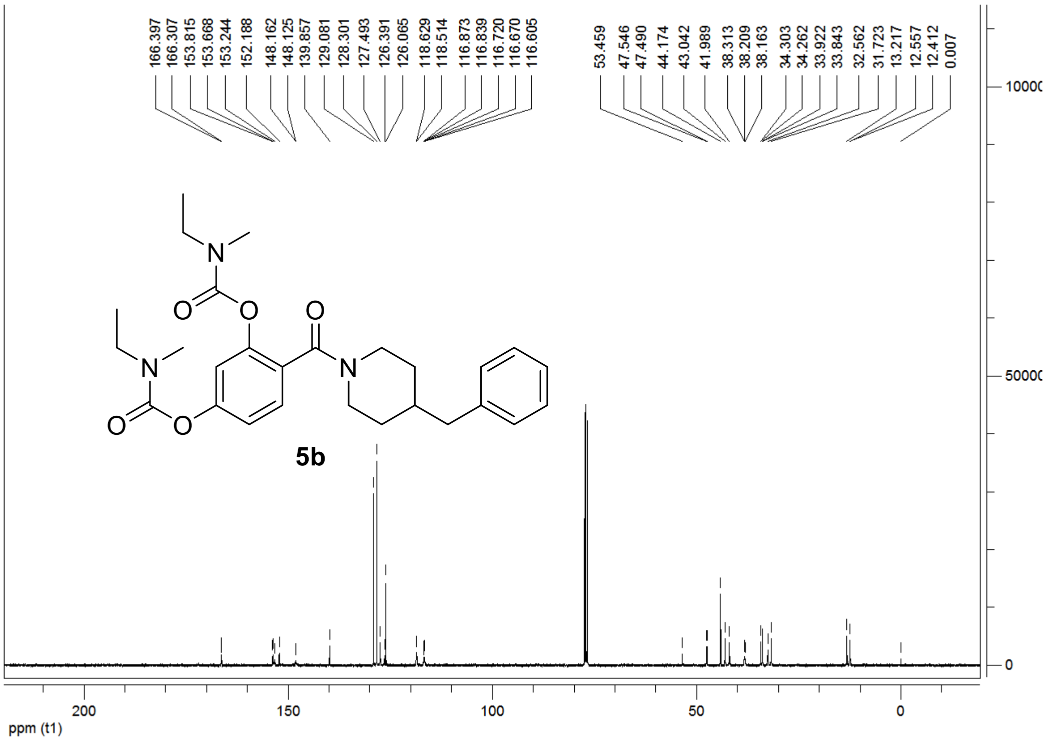

166.397
166.307
153.815
153.668
153.244
152.188
148.162
148.125
139.857
129.081
128.301
127.493
126.391
126.065
118.629
118.514
116.873
116.839
116.720
116.670
116.605
53.459
47.546
47.490
44.174
43.042
41.989
38.313
38.209
38.163
34.303
34.262
33.922
33.843
32.562
31.723
13.217
12.557
12.412
0.007
5b
ppm (t1)

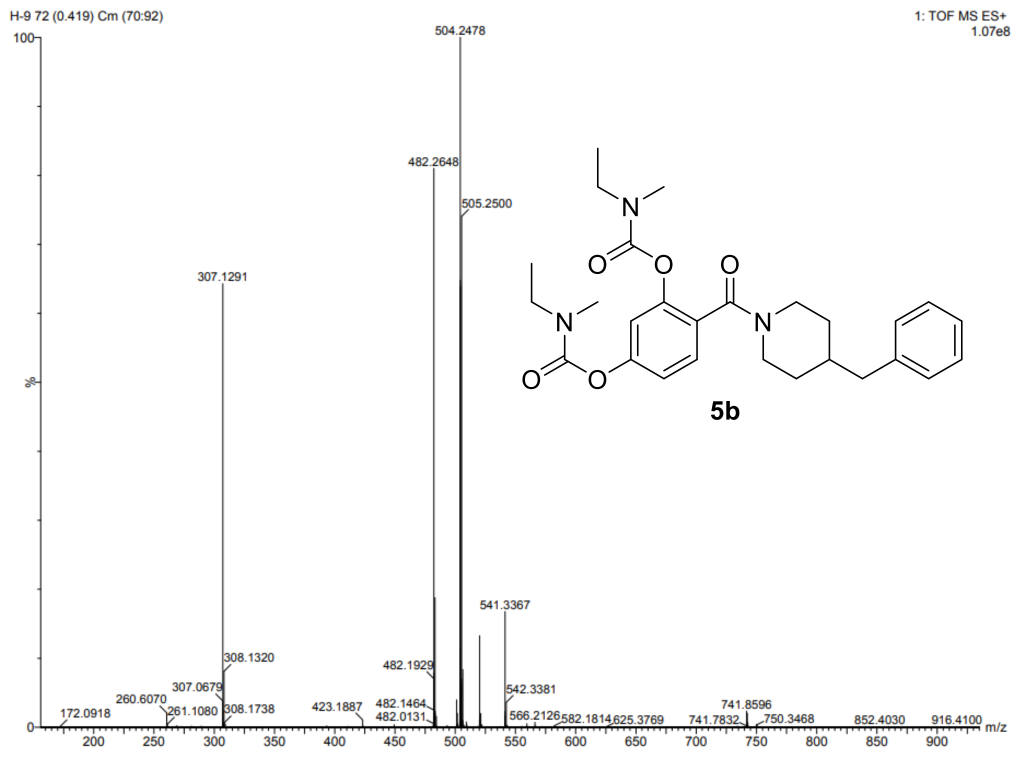

H-9 72 (0.419) Cm (70:92)
1: TOF MS ES+
1.07e8
504.2478
482.2648
505.2500
307.1291
541.3367
308.1320
482.1929
307.0679
260.6070
172.0918
261.1080
308.1738
423.1887
482.1464
482.0131
542.3381
566.2126
582.1814
625.3769
741.8596
741.7832
750.3468
852.4030
916.4100
m/z
%
5b

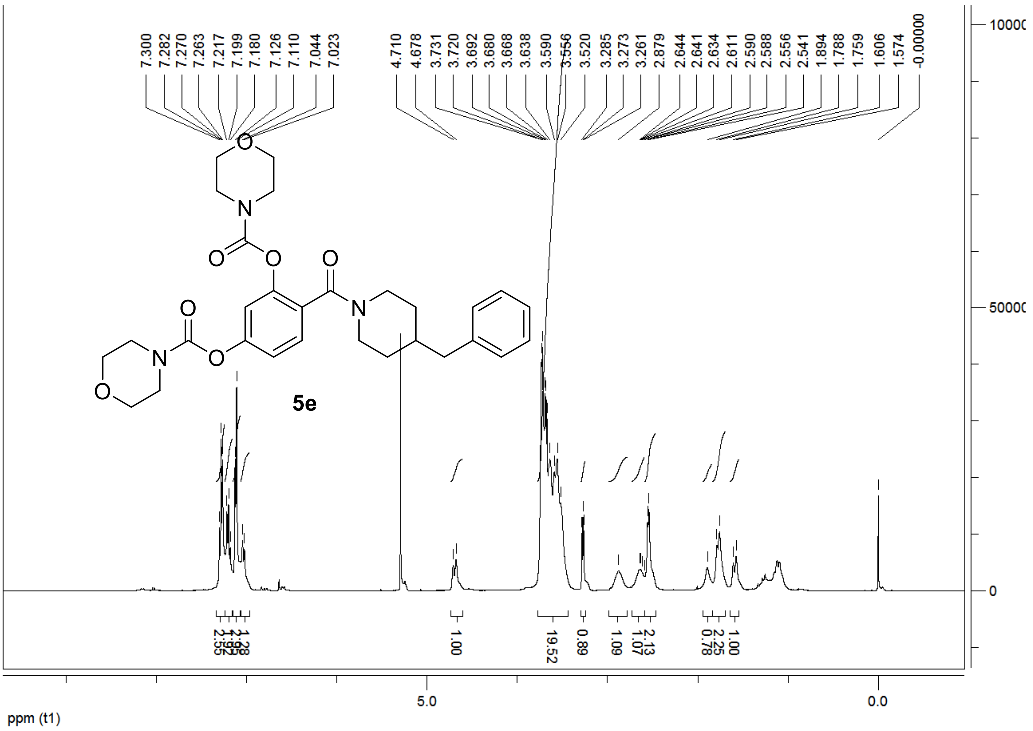
7.300
7.282
7.270
7.263
7.217
7.199
7.180
7.126
7.110
7.044
7.023
4.710
4.678
3.731
3.720
3.692
3.680
3.668
3.638
3.590
3.556
3.520
3.285
3.273
3.261
2.879
2.644
2.641
2.634
2.611
2.590
2.588
2.556
2.541
1.894
1.788
1.759
1.606
1.574
-0.00000
5e
1.00
19.52
0.89
1.09
1.07
2.13
0.78
2.25
1.00
5.0
0.0
ppm (t1)
0
50000
10000

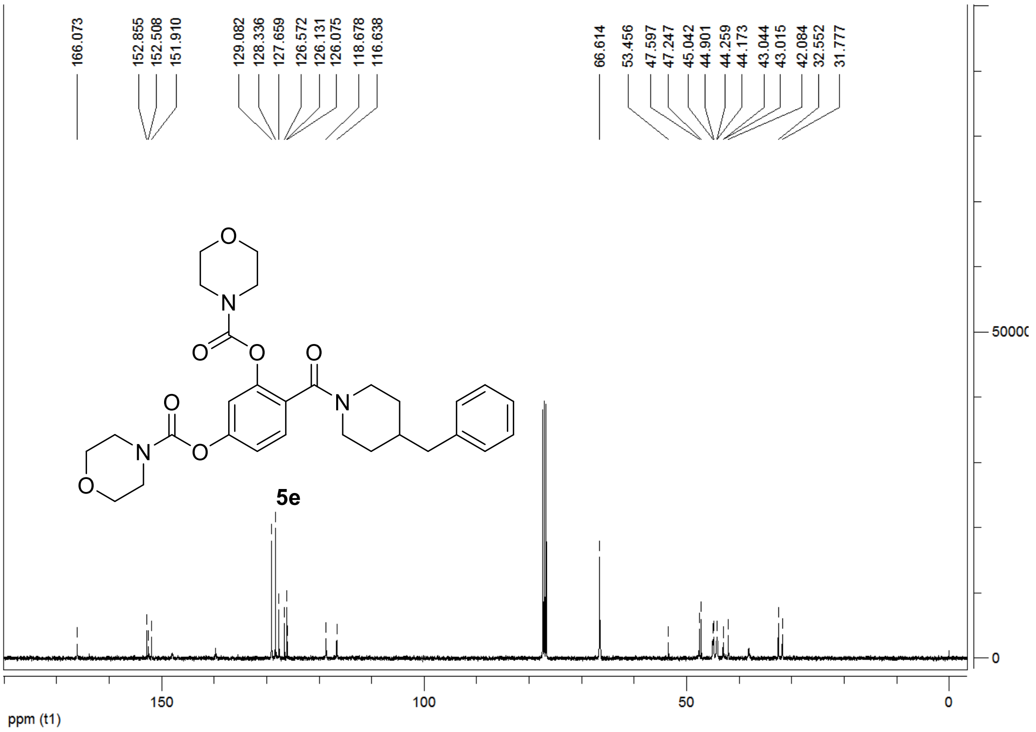
166.073
152.855
152.508
151.910
129.082
128.336
127.659
126.572
126.131
126.075
118.678
116.638
66.614
53.456
47.597
47.247
45.042
44.901
44.259
44.173
43.044
43.015
42.084
32.552
31.777
5e
150
100
50
0
ppm (t1)
0
50000

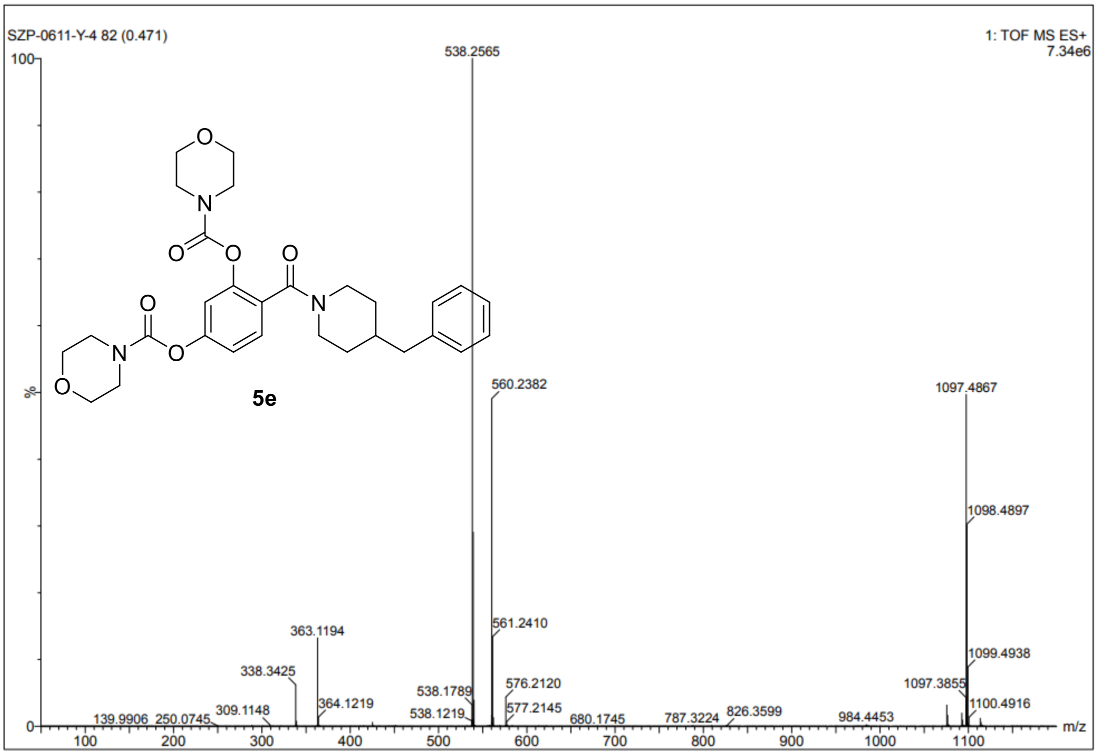
SZP-0611-Y-4 82 (0.471)
1: TOF MS ES+
7.34e6
538.2565
560.2382
1097.4867
1098.4897
561.2410
363.1194
1099.4938
338.3425
1097.3855
576.2120
538.1789
364.1219
1100.4916
309.1148
538.1219
577.2145
139.9906
250.0745
680.1745
787.3224
826.3599
984.4453
5e
%
m/z

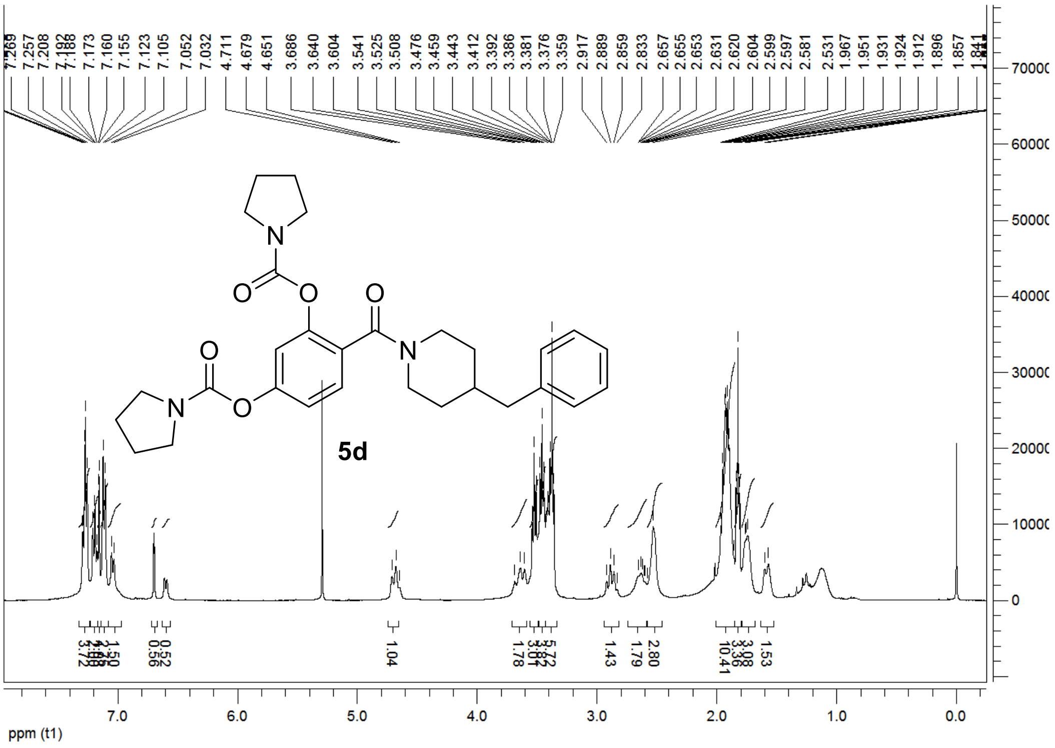
5d
ppm (t1)

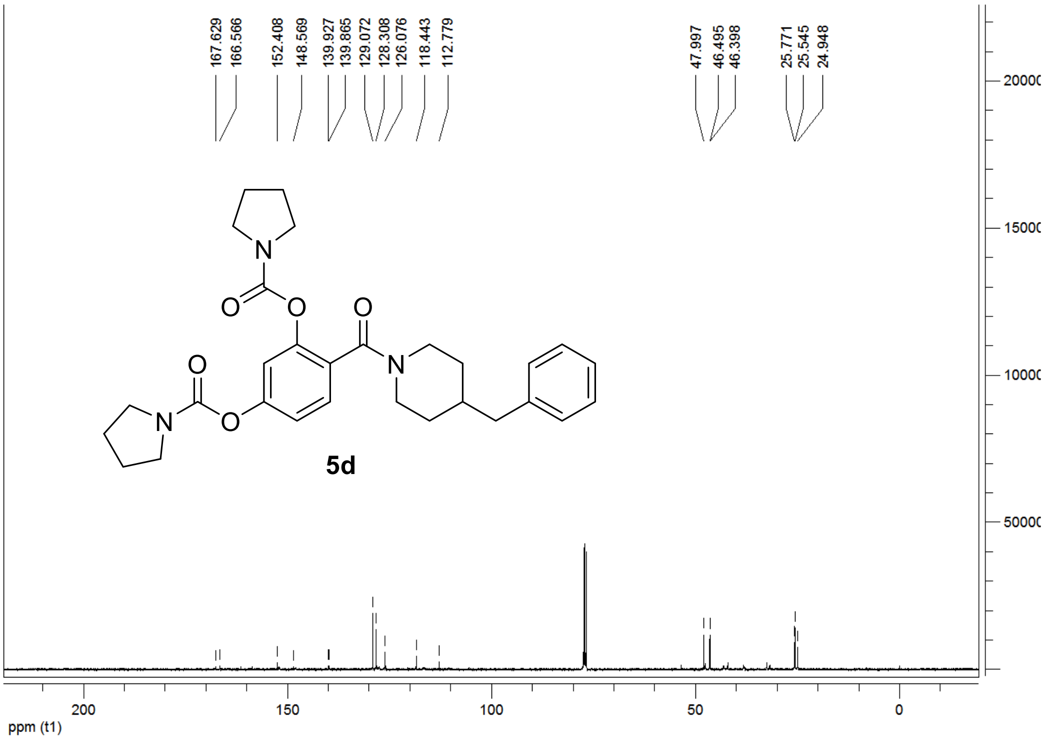
167.629
166.566
152.408
148.569
139.927
139.865
129.072
128.308
126.076
118.443
112.779
47.997
46.495
46.398
25.771
25.545
24.948
5d
ppm (t1)

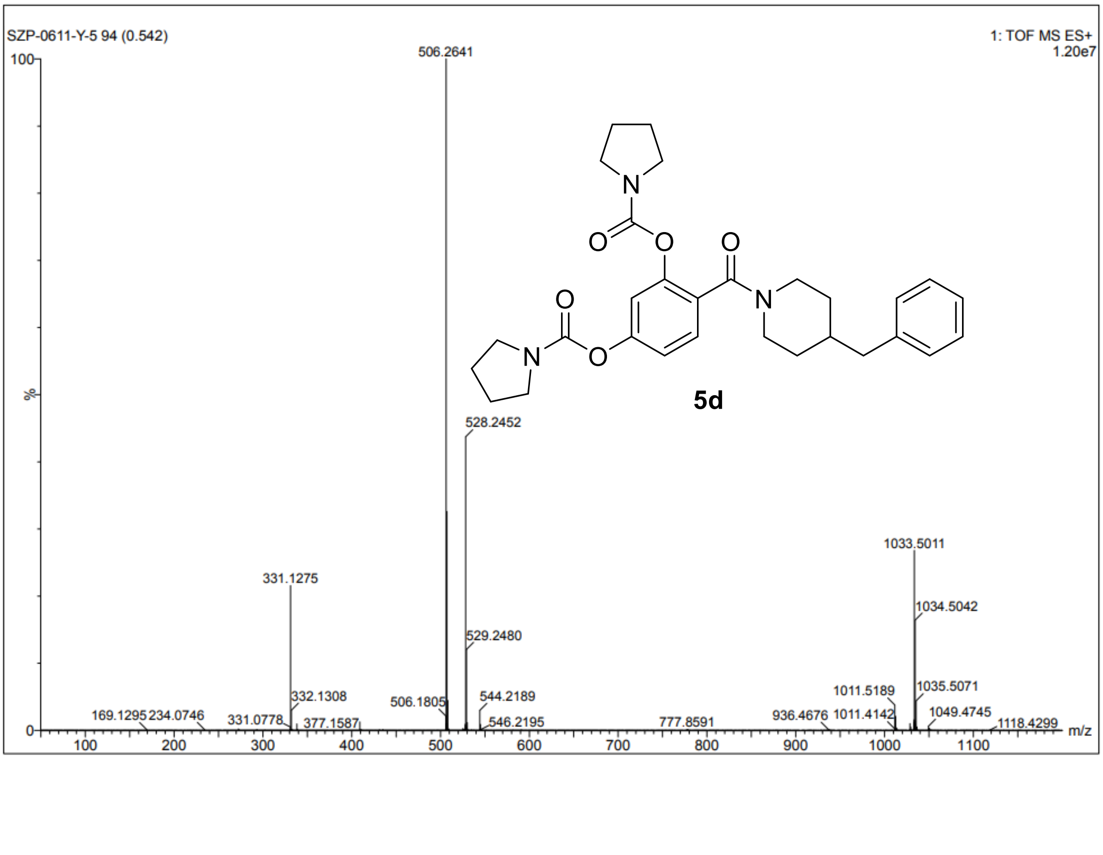
SZP-0611-Y-5 94 (0.542)
1: TOF MS ES+
1.20e7
506.2641
528.2452
331.1275
1033.5011
1034.5042
529.2480
332.1308
506.1805
544.2189
1011.5189
1035.5071
169.1295
234.0746
331.0778
377.1587
546.2195
777.8591
936.4676
1011.4142
1049.4745
1118.4299
m/z
%
5d

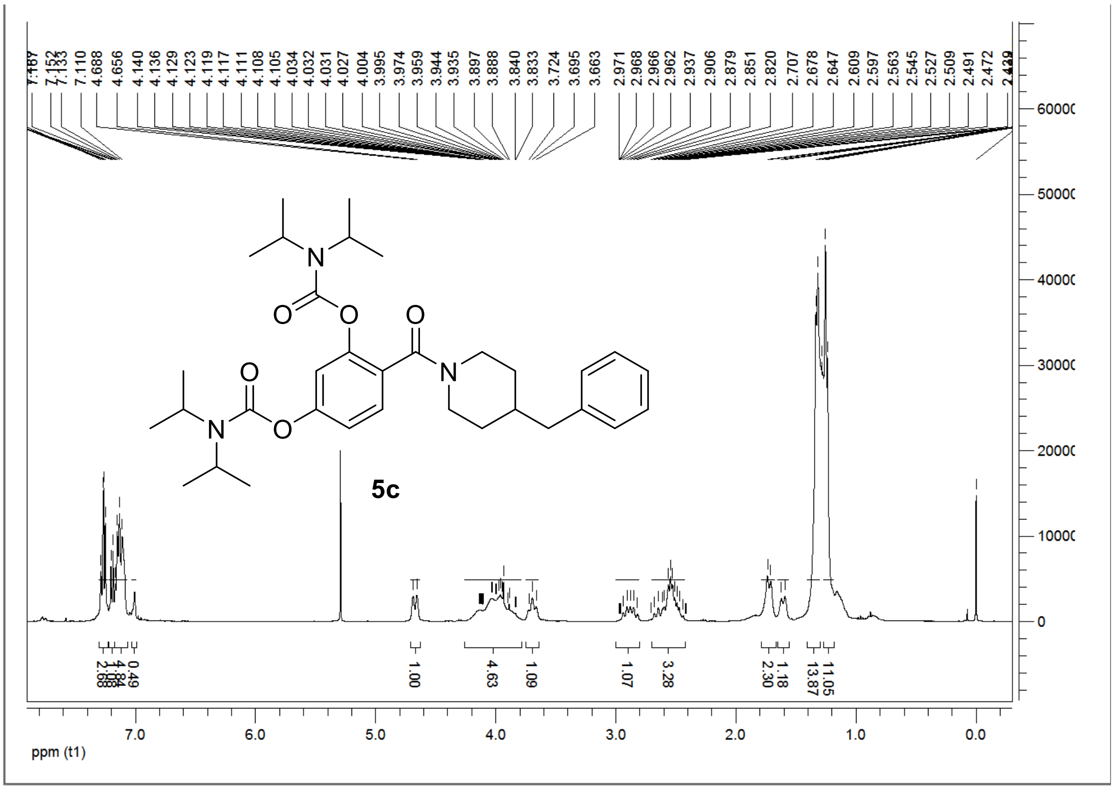
5c
ppm (t1)
7.0
6.0
5.0
4.0
3.0
2.0
1.0
0.0
60000
50000
40000
30000
20000
10000
0
7.152
7.110
4.688
4.656
4.140
4.136
4.129
4.123
4.119
4.117
4.111
4.108
4.105
4.034
4.032
4.031
4.027
4.004
3.995
3.974
3.959
3.944
3.935
3.897
3.888
3.840
3.833
3.724
3.695
3.663
2.971
2.968
2.966
2.962
2.937
2.906
2.879
2.851
2.820
2.707
2.678
2.647
2.609
2.597
2.563
2.545
2.527
2.509
2.491
2.472
0.49
1.00
4.63
1.09
1.07
3.28
2.30
1.18
13.87
11.05

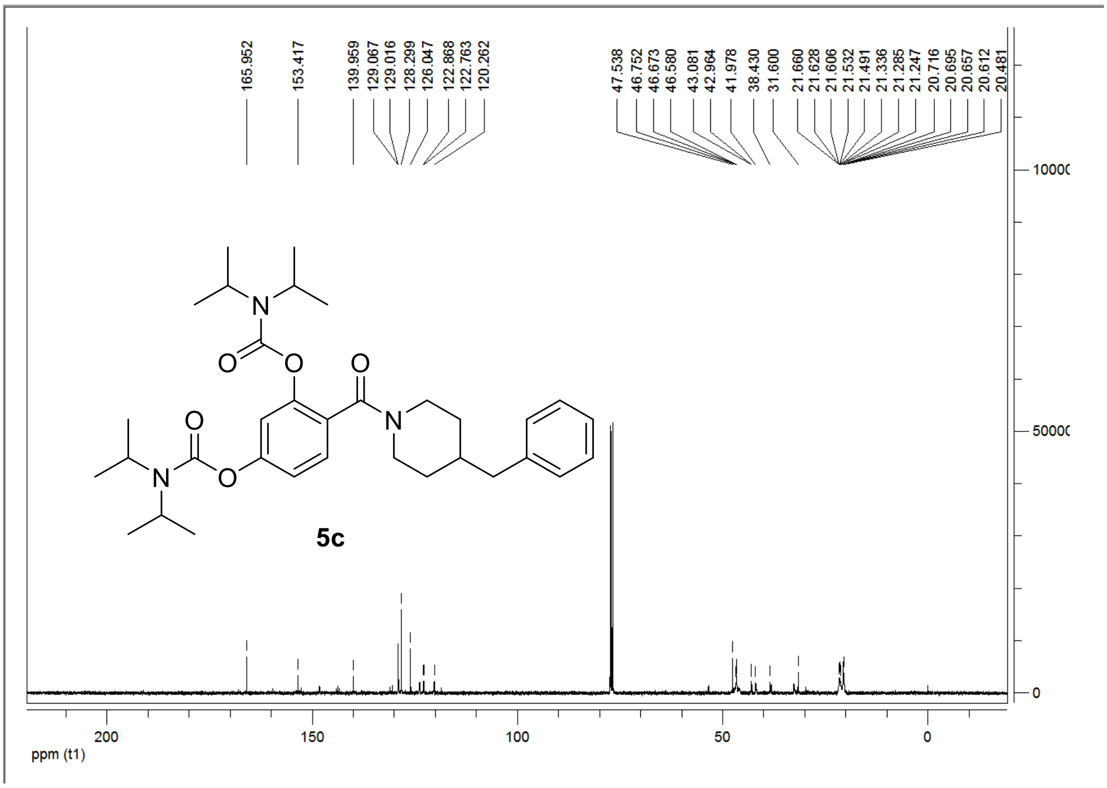
5c
ppm (t1)
200
150
100
50
0
100000
50000
0
165.952
153.417
139.959
129.067
129.016
128.299
126.047
122.868
122.763
120.262
47.538
46.752
46.673
46.580
43.081
42.964
41.978
38.430
31.600
21.660
21.628
21.606
21.532
21.491
21.336
21.285
21.247
20.716
20.695
20.657
20.612
20.481

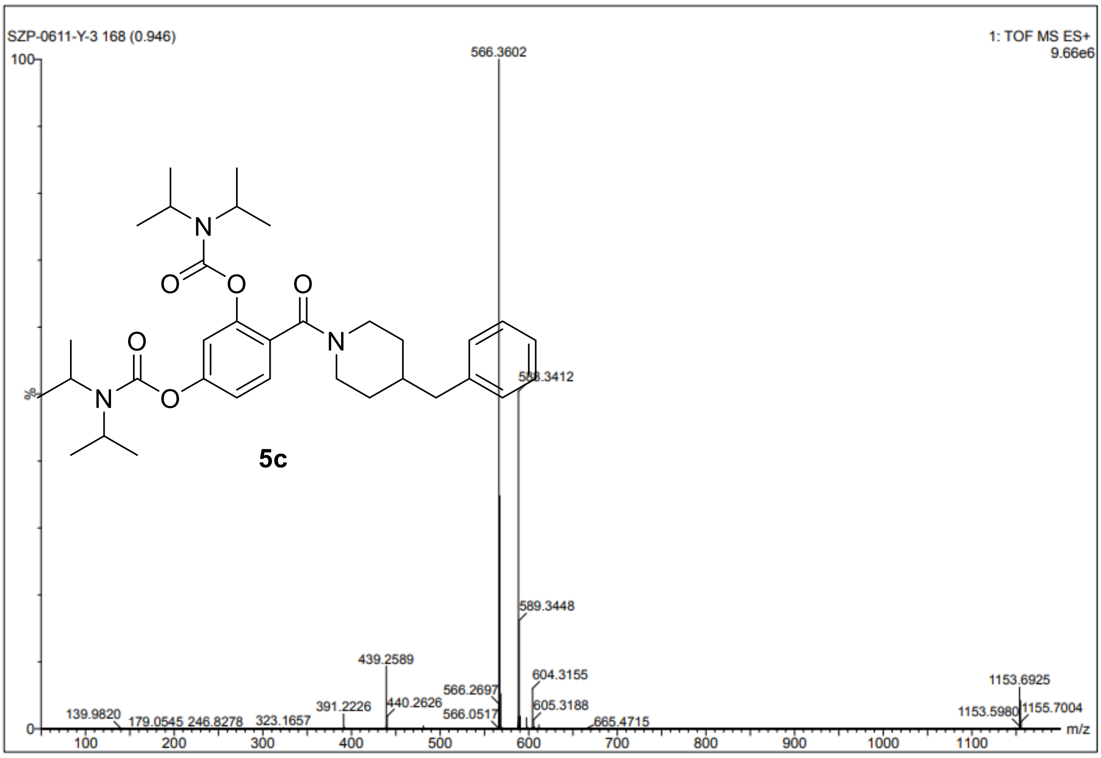
SZP-0611-Y-3 168 (0.946)
1: TOF MS ES+
9.66e6
566.3602
588.3412
589.3448
439.2589
604.3155
566.2697
440.2626
566.0517
605.3188
391.2226
139.9820
179.0545
246.8278
323.1657
665.4715
1153.6925
1153.5980
1155.7004
m/z
%
5c

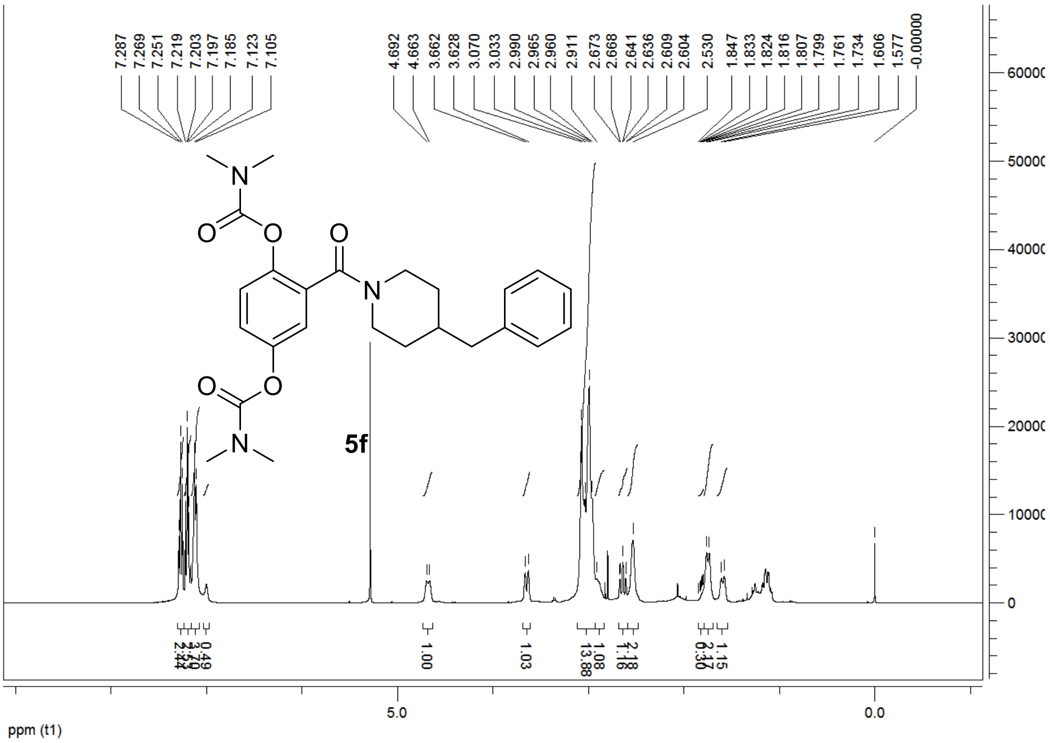
7.287
7.269
7.251
7.219
7.203
7.197
7.185
7.123
7.105
4.692
4.663
3.662
3.628
3.070
3.033
2.990
2.965
2.960
2.911
2.673
2.668
2.641
2.636
2.609
2.604
2.530
1.847
1.833
1.824
1.816
1.807
1.799
1.761
1.734
1.606
1.577
-0.00000
5f
1.00
1.03
13.88
1.08
2.18
1.15
ppm (t1)
5.0
0.0

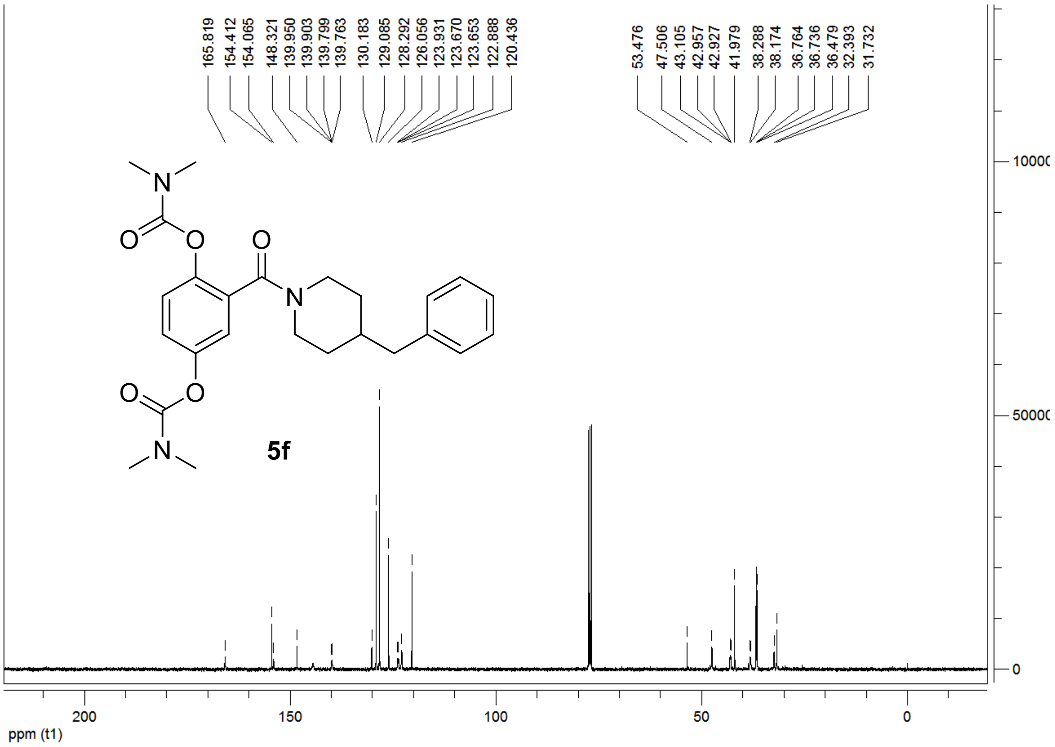

165.819
154.412
154.065
148.321
139.950
139.903
139.799
139.763
130.183
129.085
128.292
126.056
123.931
123.670
123.653
122.888
120.436
53.476
47.506
43.105
42.957
42.927
41.979
38.288
38.174
36.764
36.736
36.479
32.393
31.732
5f
200
150
100
50
0
ppm (t1)

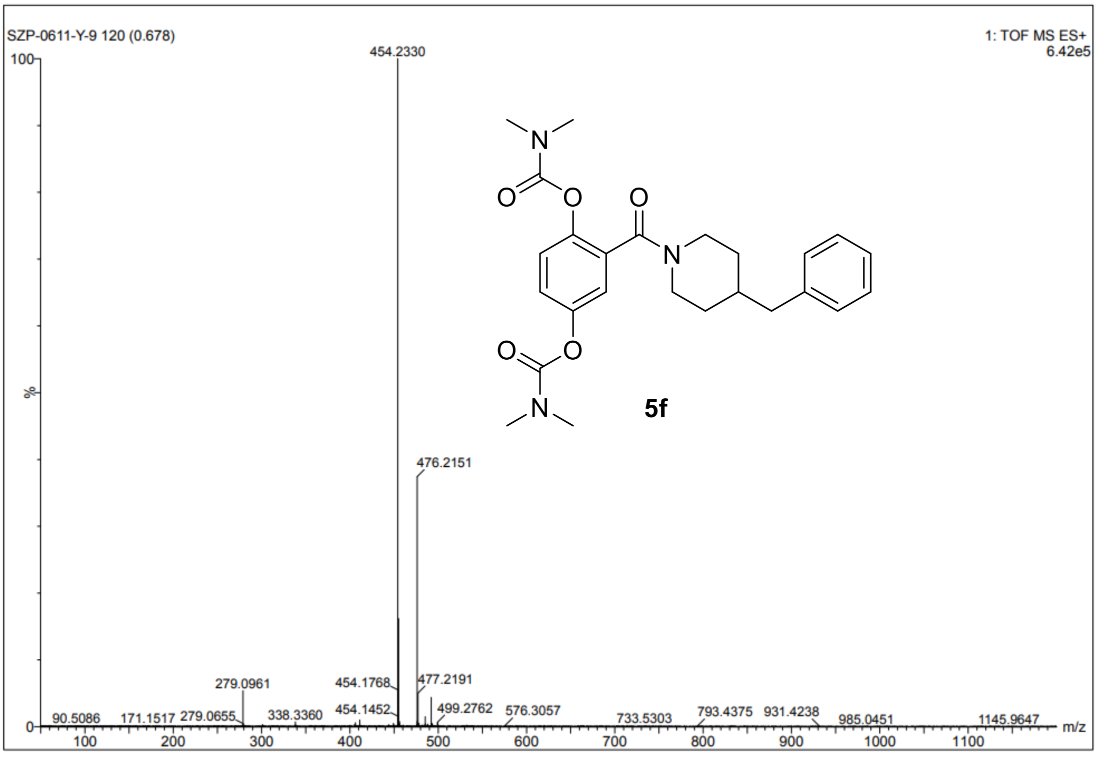

SZP-0611-Y-9 120 (0.678)
1: TOF MS ES+
6.42e5
454.2330
476.2151
279.0961
454.1768
477.2191
90.5086
171.1517
279.0655
338.3360
454.1452
499.2762
576.3057
733.5303
793.4375
931.4238
985.0451
1145.9647
m/z
%
5f

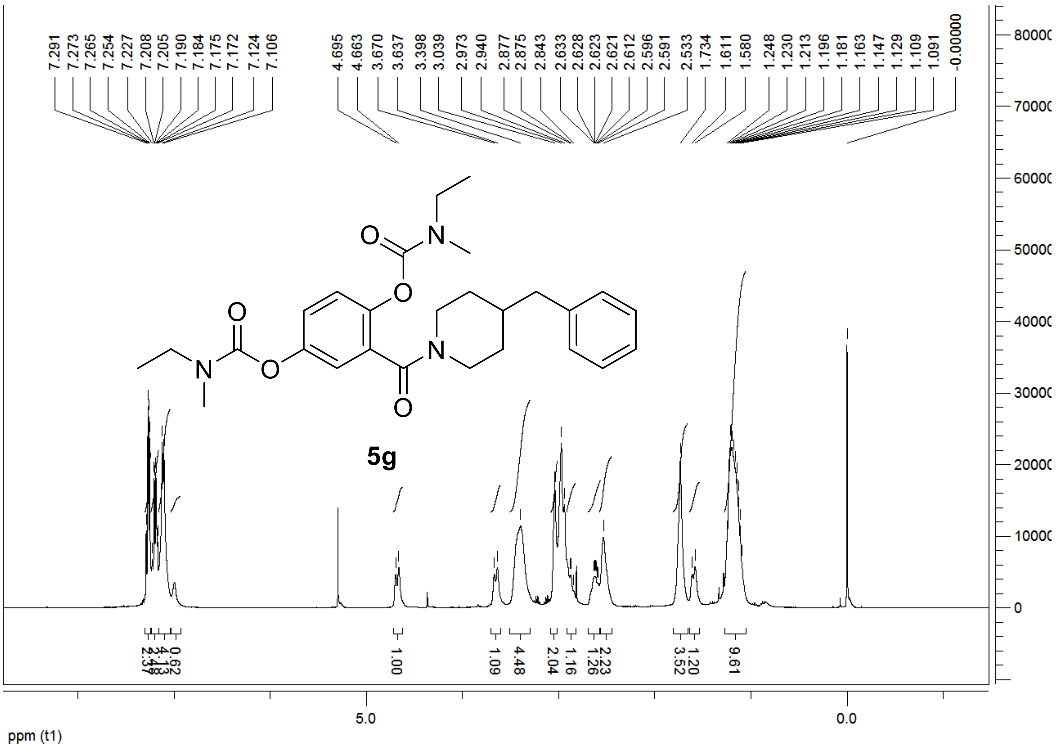

7.291
7.273
7.265
7.254
7.227
7.208
7.205
7.190
7.184
7.175
7.172
7.124
7.106
4.695
4.663
3.670
3.637
3.398
3.039
2.973
2.940
2.877
2.875
2.843
2.633
2.628
2.623
2.621
2.612
2.596
2.591
2.533
1.734
1.611
1.580
1.248
1.230
1.213
1.196
1.181
1.163
1.147
1.129
1.109
1.091
-0.00000
O
N
O
O
N
O
N
O
O
5g
1.00
1.09
4.48
2.04
1.16
1.26
2.23
3.52
1.20
9.61
0.62
5.0
0.0
ppm (t1)
80000
70000
60000
50000
40000
30000
20000
10000
0

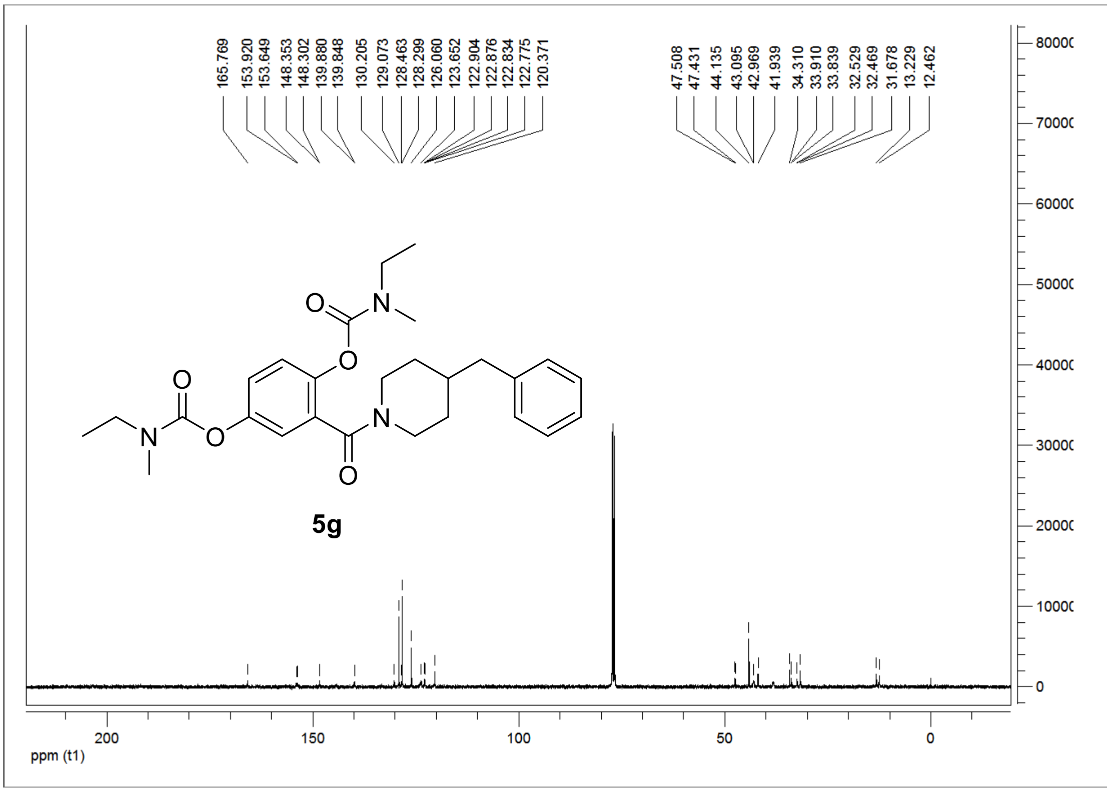

165.769
153.920
153.649
148.353
148.302
139.880
139.848
130.205
129.073
128.463
128.299
126.060
123.652
122.904
122.876
122.834
122.775
120.371
47.508
47.431
44.135
43.095
42.969
41.939
34.310
33.910
33.839
32.529
32.469
31.678
13.229
12.462
O
N
O
O
N
O
N
O
O
5g
200
150
100
50
0
ppm (t1)
80000
70000
60000
50000
40000
30000
20000
10000
0

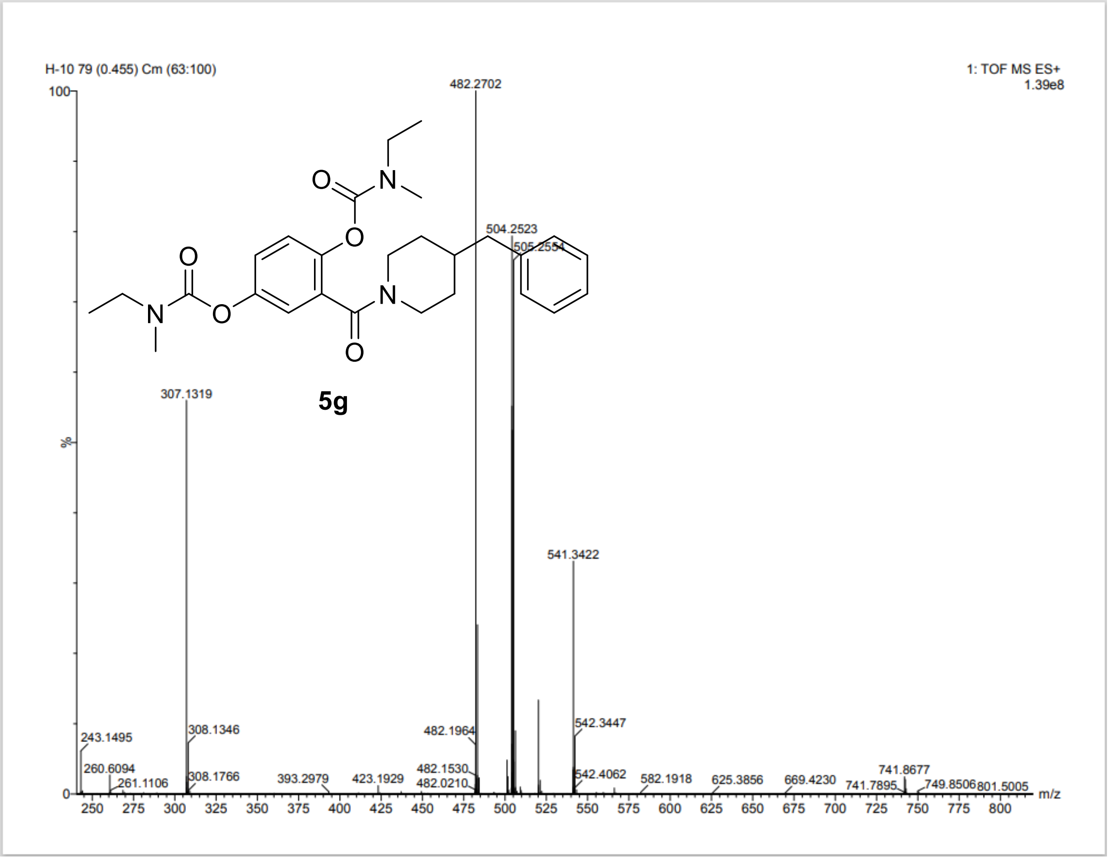
H-10 79 (0.455) Cm (63:100)
1: TOF MS ES+
1.39e8
482.2702
504.2523
505.2554
307.1319
541.3422
5g
243.1495
308.1346
482.1964
542.3447
260.6094
261.1106
308.1766
393.2979
423.1929
482.1530
482.0210
542.4062
582.1918
625.3856
669.4230
741.7895
741.8677
749.8506
801.5005
m/z

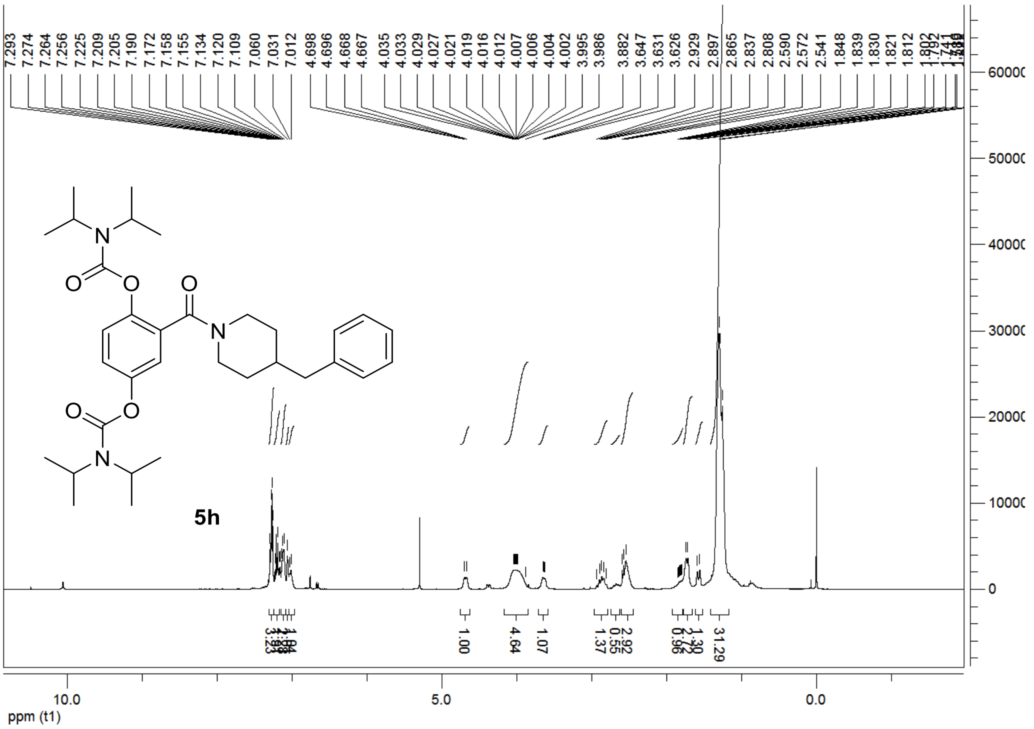
5h
ppm (t1)

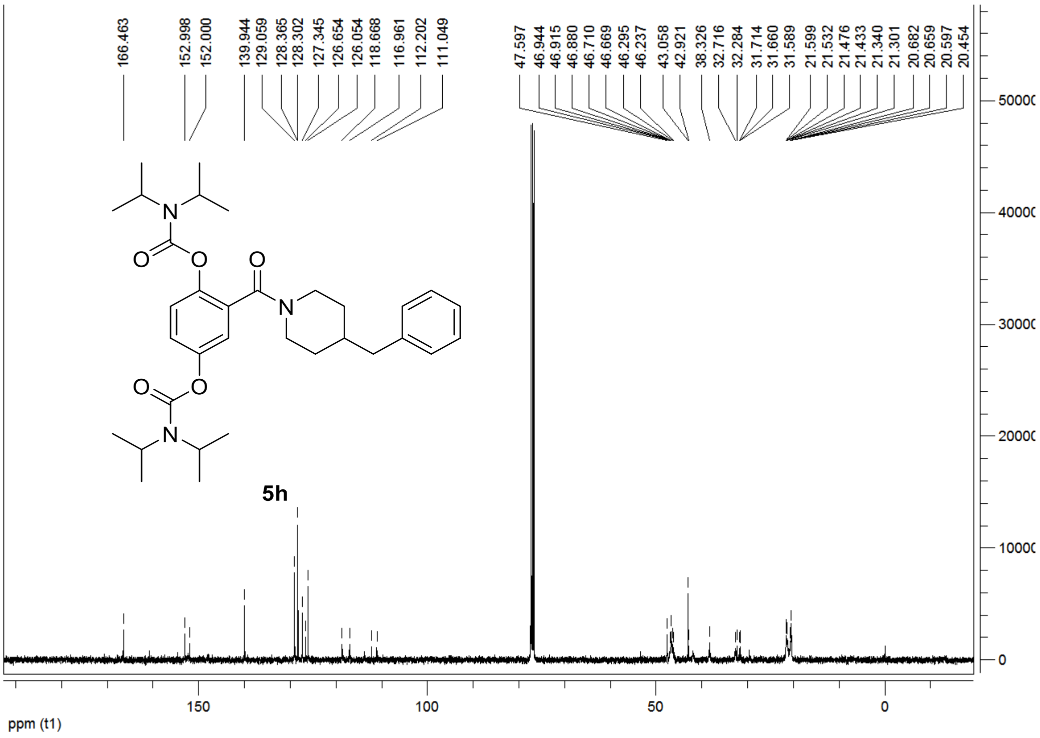

166.463
152.998
152.000
139.944
129.059
128.365
128.302
127.345
126.654
126.054
118.668
116.961
112.202
111.049
47.597
46.944
46.915
46.880
46.710
46.669
46.295
46.237
43.058
42.921
38.326
32.716
32.284
31.714
31.660
31.589
21.599
21.532
21.476
21.433
21.340
21.301
20.682
20.659
20.597
20.454
ppm (t1)

5h

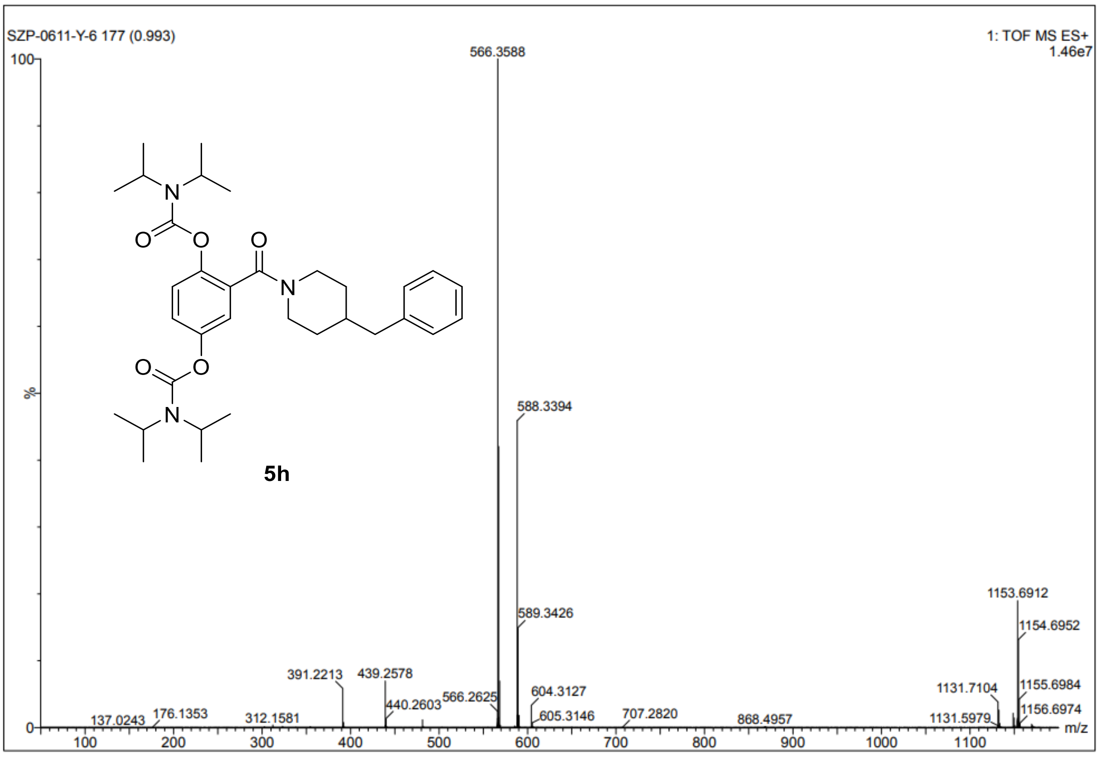

SZP-0611-Y-6 177 (0.993)
1: TOF MS ES+
1.46e7
566.3588
588.3394
589.3426
1153.6912
1154.6952
1155.6984
1156.6974
1131.7104
1131.5979
391.2213
439.2578
440.2603
566.2625
604.3127
605.3146
707.2820
868.4957
137.0243
176.1353
312.1581
%
m/z

5h
